# Feeders facilitate telomere maintenance and chromosomal stability of embryonic stem cells

**DOI:** 10.1038/s41467-018-05038-2

**Published:** 2018-07-05

**Authors:** Renpeng Guo, Xiaoying Ye, Jiao Yang, Zhongcheng Zhou, Chenglei Tian, Hua Wang, Haiying Wang, Haifeng Fu, Chun Liu, Ming Zeng, Jun Yang, Lin Liu

**Affiliations:** 1 State Key Laboratory of Medicinal Chemical Biology, Nankai University, 94 Weijin Road, Tianjin, 300071 China; 2 Department of Cell Biology and Genetics, Nankai University, 94 Weijin Road, Tianjin, 300071 China; 3 The Key Laboratory of Bioactive Materials Ministry of Education, College of Life Sciences, Nankai University, 94 Weijin Road, Tianjin, 300071 China

## Abstract

Feeder cells like mouse embryonic fibroblasts (MEFs) have been widely applied for culture of pluripotent stem cells, but their roles remain elusive. Noticeably, ESCs cultured on the feeders display transcriptional heterogeneity. We investigated roles of feeder cells by examining the telomere maintenance. Here we show that telomere is longer in mESCs cultured with than without the feeders. mESC cultures without MEF feeders exhibit telomere loss, chromosomal fusion, and aneuploidy with increasing passages. Notably, feeders facilitate heterogeneous transcription of 2-cell genes including *Zscan4* and telomere elongation. Moreover, feeders produce Fstl1 that together with BMP4 periodically activate *Zscan4*. Interestingly, *Zscan4* is repressed in mESCs cultured in 2i (inhibitors of Mek and Gsk3β signaling) media, associated with shorter telomeres and increased chromosome instability. These data suggest the important role of feeders in maintaining telomeres for long-term stable self-renewal and developmental pluripotency of mESCs.

## Abstract (web summary)

Feeder cells are widely used for the culture of embryonic stem cells (ESCs), but their specific effects are not well known. Here, the authors demonstrate that mouse ESCs exhibit telomere loss and chromosomal aberrations associated with reduced Zscan4 with increasing passages in the absence of feeders

## Introduction

Pluripotent mouse embryonic stem cells (ESCs) were originally derived and stably maintained on feeder cells such as inactivated mouse embryo fibroblasts^1^, and can generate complete ESC-pups by tetraploid embryo complementation (TEC), the most stringent functional test of naive pluripotency^2–4^. Feeders also have been widely used in maintenance of pluripotent stem cells in other species, including human and monkey^5,6^. Yet, mouse ESC cultures on feeders exhibit heterogeneity in transcription of pluripotency genes^7–9^, and notably intermittently (~1–5% of cell population) express 2-cell embryo-like (2C) genes including endogenous retroviruses, and *Zscan4* that is known to effectively elongate telomeres by recombination^10,11^. In addition, serum-based culture conditions also contribute to global transcription heterogeneity in mouse ESCs^12,13^.

Telomeres are repetitive nucleotide sequences at the end of chromosomes that protect chromosomes from deterioration or fusion, and the telomere length primarily is maintained by telomerase^14,15^. Indeed, telomerase is important for telomere elongation of ESCs and induced pluripotent stem cells (iPSCs). Haploinsuficiency or loss of telomerase limits telomere elongation of ESCs/iPSCs^16–20^. On prolonged growth, mTert-deficient ESCs exhibit genomic instability, aneuploidy and telomeric fusions^18^. Also, recombination-based alternative lengthening of telomere (ALT)-like pathways are activated to elongate telomeres to sufficient lengths required for unlimited self-renewal, genomic stability, and pluripotency of mouse ESCs/iPSCs (review^21^).

Feeder-free cultures also have been explored to sustain self-renewal of ESCs^22^. Remarkably, 2i (inhibitors of Mek and Gsk3β signaling) medium with LIF in the absence of feeders was developed to achieve ground state of mouse ESCs^23^, and also has been successfully used for derivation of germline competent ESCs in other species such as rat^24^. Notably, 2i culture gives rise to transcriptional profiles and epigenetic landscapes quite distinct from serum-based ESCs^25^, and represses or reduces the heterogeneity of expression of pluripotency genes^9,26^. Also, signaling pathways and transcriptional regulation of conventional ESCs originally derived in the presence of irradiated fibroblasts and serum differ from those of ground-state ESCs maintained in 2i media^27^.

We revisit the function and potential signaling of feeders in maintenance of telomeres and unlimited self-renewal capacity of mESCs. We find that heterogeneity in the expression of pluripotency genes and 2C-genes in ESC cultured with feeders is linked to telomere maintenance and chromosomal stability and developmental pluripotency. Feeders provide signaling such as BMP4 and Fstl1 that can enhance sporadic *Zscan4* expression that is associated with telomere maintenance and long-term self-renewal of mESCs. ESCs cultured without feeders exhibit reduced *Zscan4* expression and increased telomere signal-free ends, indicative of shortest telomere, and even chromosome fusion after extended passages. 2i condition suppresses *Dnmt3a/b* and *Zscan4* and impairs telomere maintenance and chromosomal stability of ESCs after long-term culture.

## Results

### Feeders maintain telomeres and genomic stability of ESCs

**Fig. 1:**
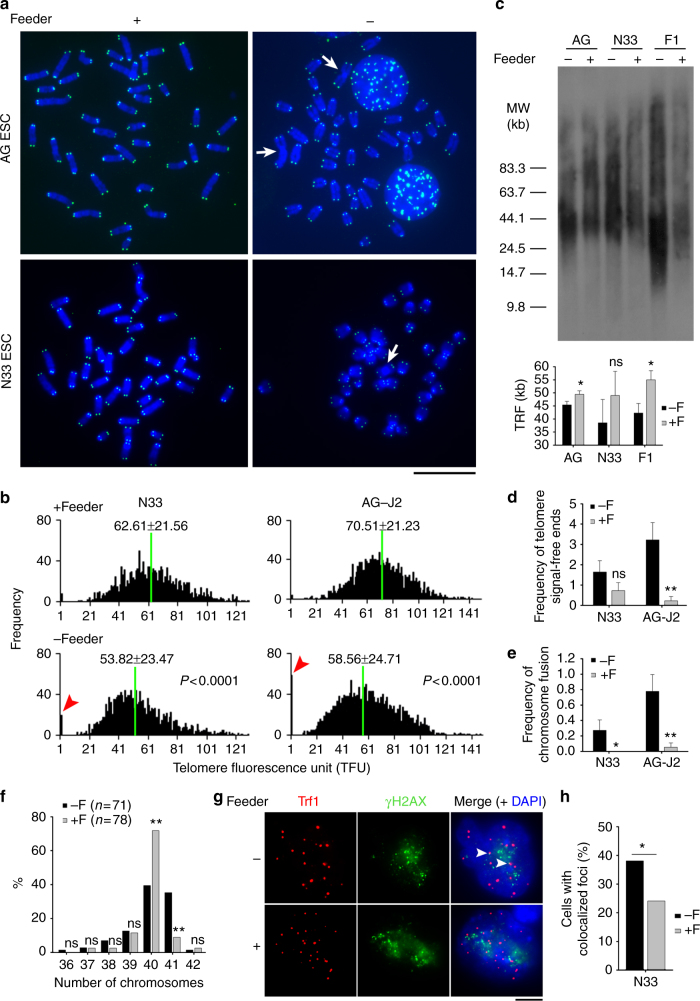
Feeders maintain telomeres of ESCs. **a** Representative telomere Q-FISH images of N33 and AG-J2 ESCs cultured on gelatin or feeder. blue, chromosomes stained by DAPI; green dots, telomeres; white arrows indicate chromosome fusion. Scale bar, 10 μm. **b** Histogram shows distribution of relative telomere length displayed as TFU by Q-FISH analysis. Green line indicates medium telomere length. Mean ± s.d. of telomere length is shown above each panel. Heavy black bars on *Y*-axis show the frequency of telomere signal-free ends. Telomere length was measured at passage 8 for N33 ESCs and passage 20 for AG-J2 ESCs under feeder or feeder-free conditions. P values were calculated by Wilcoxon–Mann–Whitney rank sum test. **c** Telomere length distribution shown as TRF by Southern blot analysis of N33 ESCs at passage 10, F1 ESCs at passage 21 and AG ESCs at passage 5 cultured with or without feeders. **d** Frequency of telomere signal-free ends indicative of shortest telomere per metaphase. **e** Frequency of chromosome fusion per metaphase. Mean ± s.e.m. **f** Distribution of chromosome numbers in ESCs cultured with or without feeders for 8–10 passages. *n* = number of spread analyzed. **g** Immunofluorescence images of Trf1 (red) and γH2AX (green). Colocalized foci are indicated by arrowheads. Scale bar, 5 μm. **h** Percentage of ESCs with colocalized foci of Trf1 and γH2AX. *n* = 100 nuclei counted from the images captured in **g**. *P < 0.05, **P < 0.01, ns not significant (*P* > 0.05). Student’s *t*-test for **c**–**e**, and *χ*^2^ test for **f** and **h**

To determine the roles of feeders on telomere maintenance, mouse ESCs were cultured on inactivated MEFs served as feeder layers (+F,) or on gelatin-coated plates without feeders (−F). LIF was added under all conditions to prevent differentiation. By telomere quantitative fluorescent in situ hybridization (Q-FISH) analysis, telomeres were longer in ESCs cultured on feeders than those without feeders in four independent ESC lines tested (Fig. 1a, b; Supplementary Fig. 1a, b; Supplementary Fig. 2a). Shorter telomeres of ESCs cultured in the absence of feeders were also revealed by Southern blot analysis, which measures telomere terminal restriction fragment (TRF) (Fig. 1c). Telomere lengths differed more in ESCs with increasing passages in culture. Moreover, frequency of telomere signal-free ends, indicative of shortest telomeres, and chromosome fusion increased in the absence of feeders (Fig. 1d, e). Further, ESCs cultured on the feeders had normal karyotypes (2*n* = 40) in the majority of spread (71.8%), in contrast to reduced (39.4%) normal karyotypes and more spread with 41 chromosomes (35.2%) in ESCs cultured without feeders after 8–10 passages (Fig. 1f). These data show that aneuploidy is increased in ESCs cultured without feeders. Telomere dysfunction-induced foci (TIFs), shown as co-localized foci of Trf1 and γH2AX^28^, were reduced in cultures with feeders (Fig. 1g, h). These data suggest that feeders play a role in maintaining telomere and genomic integrity of mouse ESCs.

**Fig. 2:**
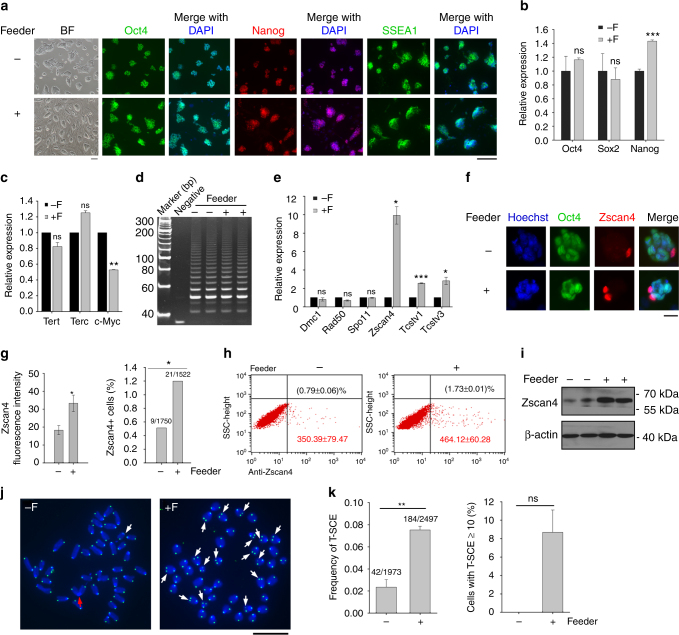
Feeders increase *Zscan4* expression and T-SCE in ESCs. **a** Morphology of F1 ESCs cultured in feeder or feeder-free conditions under bright field (BF) with phase-contrast optics and immunofluorescence of pluripotency-associated markers Oct4, Nanog, and SSEA1. Scale bar, 100 μm. **b** Expression by qPCR analysis of *Oct4*, *Sox2*, and *Nanog* in F1 ESCs cultured without (−F) or with feeders (+F). **c** Expression of telomerase-related genes *Tert*, *Terc*, and *c-Myc* in −F and +F ESCs. **d** Telomerase activity measured by TRAP assay. Lysis buffer served as negative control. **e** Expression by qPCR of genes for telomere recombination, *Dmc1*, *Rad50*, *Spo11*, and 2C genes, *Zscan4*, *Tcstv1*, and *Tcstv3*. **f** Immunofluorescence co-staining of Oct4 and Zscan4 shows Zscan4-positive cells mutually exclusive of Oct4. Scale bar, 20 μm. **g** Relative Zscan4 fluorescence intensity estimated by ImageJ and percentage of Zscan4^+^ cells (number of ESCs counted). **h** Flow cytometry quadrantal diagram indicates percentage and mean fluorescence intensity of Zscan4^+^ cells in ESCs. **i** Western blot analysis of Zscan4 in −F and +F ESCs. **j** Representative CO-FISH images showing T-SCE (indicated by white arrows) in −F and +F ESCs. Red arrow, chromosome fusion. Scale bar, 10 μm. **k** Frequency of T-SCE (total T-SCE/total chromosomes) and percentage of ESCs with T-SCE foci ≥10 in −F and +F ESCs. **c**–**k** N33 ESCs. Mean ± s.e.m. from three independent experiments. *P < 0.05, **P < 0.01, ***P < 0.001, ns not significant (*P* > 0.05). *χ*^2^ test for **g** (right) and **k**, and Student’s *t*-test for **b**, **c**, **e**, **g** (left). −F without feeders, +F with feeders

However, expression levels of important pluripotent marker genes, including *Oct4*, *Sox2*, *Nanog*, *Utf1* measured by quantitative real-time PCR (qPCR) and Oct4, Nanog, SSEA1 determined by immunofluorescence microscopy, did not differ between ESCs cultured with and without feeders (Fig. 2a, b; Supplementary Fig. 3a–c), except for slightly increased expression of *Nanog* in F1 ESCs cultured with feeders (Fig. 2b). E-cad-Fc as feeder-free culture condition reportedly maintains pluripotent ESCs without colony formation^29^. ESCs cultured on E-cad-Fc expressed pluripotent genes at levels similar to those of feeder-free with gelatin only or feeder conditions (Supplementary Fig. 3a, b). These data indicate that telomere shortening in ESCs cultured under feeder-free conditions is not likely caused by cell differentiation.

To test the developmental pluripotency, we injected ESCs into four- to eight-cell embryos to evaluate their competence in generating chimeric mice (see Methods). ESCs cultured in the presence of feeders produced chimeras at high efficiency (75%), in contrast to those without feeders (12.5%) that had no all black pups (Supplementary Fig. 1c; Supplementary Table 1). Contributions of ESCs to various tissues and germline of the chimeras were confirmed by microsatellite genotyping (Supplementary Fig. 1d, e). Collectively, ESCs cultured with feeders exhibit naive developmental pluripotency as evidenced by high germline competency, whereas feeder-free ESCs show reduced developmental pluripotency.

### Feeders facilitate *Zscan4* expression and T-SCE

To investigate the mechanisms underlying telomere dysfunction in ESCs culture under feeder-free condition, we analyzed expression of key telomerase genes *Tert*, *Terc*, and *Tert* positive regulator *c-Myc*. Relative expression levels of *Tert* and *Terc* were similar between ESCs cultured with and without feeders (Fig. 2c; Supplementary Fig. 2b). c-Myc expression was reduced in N33 ESCs without feeders, but not in feeder-free J1 ESCs (Fig. 2c; Supplementary Fig. 2b). Telomerase activity did not differ between ESCs with and without feeders (Fig. 2d; Supplementary Fig. 2c). Telomerase extends the telomeres at a slow pace of about 50–100 nucleotides per cell cycle^30^. But telomeres elongate more efficiently (>5 kb) within only 5–8 passages in ESCs cultured with feeders (Fig. 1a–c), suggesting that mechanisms other than telomerase also are involved in telomere elongation of ESCs cultured with feeders.

*Zscan4* is responsible for rapid telomere elongation by recombination-based mechanism^11^. We tested whether feeders can activate *Zscan4*^*+*^/2C-like state of ESCs. Levels of *Zscan4* increased ~6–10-fold in two independent ESC lines cultured on feeders compared with those without feeders (Fig. 2e; Supplementary Fig. 2d). Levels of *Tcstv1* and *Tcstv3* were also increased in ESCs cultured with feeders, while expression levels of other three genes associated with telomere recombination, *Dmc1*, *Spo11, Rad50*^11^, did not differ between ESC cultures with and without feeders (Fig. 2e; Supplementary Fig. 2d). By immunofluorescence, Zscan4 was sporadically expressed and incompatible with Oct4 expression (Fig. 2f). Relative Zscan4 fluorescence and the proportion of Zscan4^+^ cells were elevated in ESCs cultured on feeders (Fig. 2g, h; Supplementary Fig. 2e). Protein levels of Zscan4 also were increased in ESCs cultured on feeders (Fig. 2i; Supplementary Fig. 2f). Of the naive genes *Klf4*, *Stella*, *Rex1*, and *Tbx3, Klf4* and *Tbx3* with heterogeneous expression pattern were upregulated in ESCs cultured on the feeder (Supplementary Fig. 2g–i).

We assessed telomere-sister chromatid exchange (T-SCE) in ESCs by telomere chromosome orientation FISH (CO-FISH) analysis. Frequency of T-SCE was elevated in ESCs cultured on the feeders (Fig. 2j, k). Notably, high frequency of T-SCE (T-SCE ≥ 10 foci/cell) was exclusively emerged in ESCs cultured with feeders (Fig. 2j, k), presumably representative of undergoing telomere elongation mediated by Zscan4. Taken together, feeders increase the population of ESCs that enter *Zscan4*^+^/2C-like state and promote T-SCE.

**Fig. 3:**
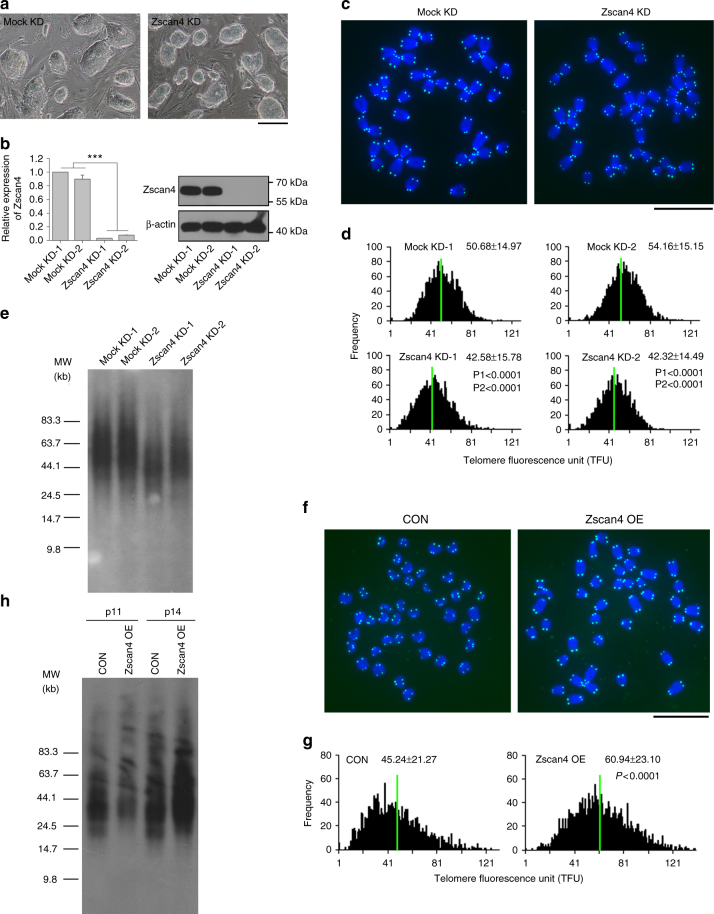
Knockdown of *Zscan4* leads to telomere shortening and overexpression of *Zscan4* elongates telomeres. **a** Colony morphology of control and *Zscan4* knockdown (KD) J1 ESCs cultured on feeders, Scale bar, 50 μm. **b** Depletion of *Zscan4* validated by qPCR and western blot after stable *Zscan4* KD for 12 passages. Mean ± s.e.m. ***P < 0.001. Student’s *t*-test. **c** Representative telomere Q-FISH images of control and *Zscan4* KD ESCs. Blue, chromosomes stained by DAPI; green dots, telomeres. Scale bar, 10 μm. **d** Histogram shows distribution of relative telomere length displayed as TFU by Q-FISH analysis. Green line indicates medium telomere length. Mean ± s.d. of telomere length is shown above each panel. P1, compared with Mock KD-1, P2, compared with Mock KD-2. Wilcoxon–Mann–Whitney rank sum test. **e** Telomere length distribution shown as TRF by Southern blot analysis. **f** Representative telomere Q-FISH images of control and *Zscan4* overexpression (OE) J1 ESCs cultured without feeders. Scale bar, 10 μm. **g** Histogram shows distribution of relative telomere length displayed as TFU by Q-FISH analysis. Wilcoxon–Mann–Whitney rank sum test. **h** Telomere length distribution shown as TRF by Southern blot analysis of control and *Zscan4* OE ESCs at two indicated passages (P11 and P14)

To examine the effect of *Zscan4* on telomere maintenance, we depleted *Zscan4* by shRNA in ESCs cultured on the feeders (Fig. 3a, b). Telomeres shortened in ESCs depleted of *Zscan4* (Fig. 3c–e). On the contrary, forced expression of *Zscan4* in ESCs cultures without feeders elongated telomeres compared with controls (Fig. 3f–h). These data suggest that Zscan4 can regulate telomere length in ESCs cultured on the feeders.

### Transcriptome profile and signaling pathways by feeders

**Fig. 4:**
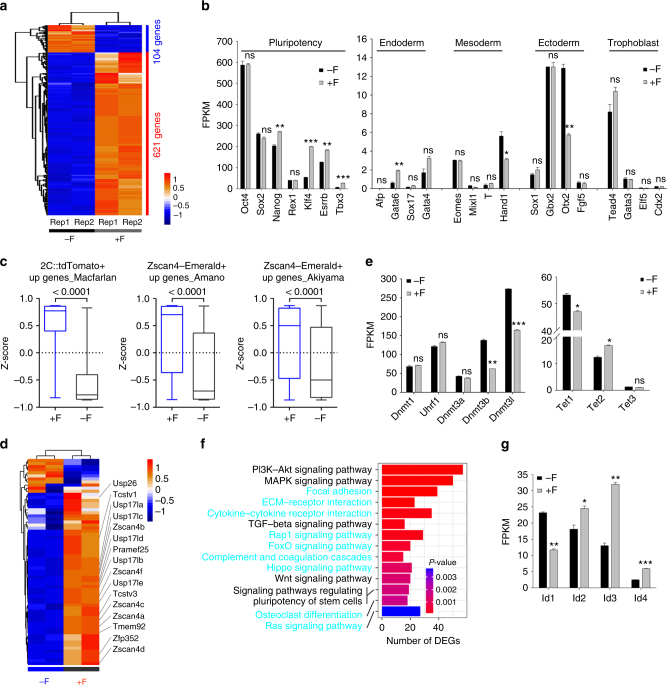
Transcriptome and signal pathways by RNA-seq analysis. **a** Heatmap illustrating differentially expressed genes (DEGs) between N33 ESCs cultured without (−F) and with feeders (+F). Two biological replicates were analyzed per group. Genes with ≥2-fold expression changes, *P*adj < 0.05 were chosen for heatmap. **b** Expression levels (FPKMs) of pluripotency and lineage marker genes in −F and +F ESCs. **c** *Z*-scores of several gene lists: upregulated genes in 2C::tdTomato^+^ ESCs from Macfarlan et al.^10^, Zscan4-Emerald + ESCs from Amano et al.^31^, or in Zscan4-Emerald + ESCs from Akiyama et al.^32^. **d** Heatmap shows expression of representative 2C genes in −F and +F ESCs. **e** Expression levels (FPKMs) of multiple genes involved in DNA (de)methylation. **f** The enriched KEGG pathways of DEGs between −F and +F ESCs. **g** Expression levels (FPKMs) of BMP-Smad targeting Id genes. *P < 0.05, **P < 0.01, ***P < 0.001, ns not significant (*P* > 0.05). Student’s *t*-test

To understand signal pathways and mechanisms underlying *Zscan4* expression in ESCs cultured on feeders, we performed RNA-seq analysis of the transcriptome of ESCs cultured with or without feeders. Compared with those of feeder-free ESCs, 621 genes were upregulated and only 104 genes downregulated in ESCs cultured with feeders (Fig. 4a, *P*adj < 0.05, fold change ≥ 2), suggesting higher transcriptional activity in ESCs cultured with feeders. Consistent with qPCR data shown above, expression levels of pluripotency genes *Oct4*, *Sox2*, and *Rex1* were similar between ESCs cultured with and without feeders, but *Nanog*, *Klf4, Esrrb,* and *Tbx3* were upregulated in ESC culture with feeder (Fig. 4b). Representative lineage marker genes expressed at quite low levels overall (based on low FPKMs) in ESCs cultured with or without feeders, compared with those of pluripotency genes. Endoderm marker genes *Gata4* and *Gata6* were increased in ESCs with feeders, while *Hand1* in mesoderm and also in trophectoderm lineage and *Otx2* in the ectoderm upregulated in ESCs without feeders (Fig. 4b).

We took advantages of previously reported lists of up-regulated genes in two-cell (2C)-like Zscan4^+^ ESCs compared to Zscan4^−^ ESCs to evaluate expression of 2C genes in ESCs cultured with or without feeders^10,31,32^. Consistently, ESCs cultured on feeders showed significantly higher Z-scores than those without feeders in three independent gene lists (Fig. 4c). Representative 2C genes, e.g., *Zscan4*, *Tcstv1/3*, *Usp17l*, *Pramef25*, *Tmem92*, were enriched in ESCs cultured with feeders (Fig. 4d). These data together indicate notably activated *Zscan4*^+^/2C-like state in ESCs with feeders. Genes involved in de novo DNA methylation, *Dnmt3b* and *Dnmt3l*, were downregulated in ESCs with feeders (Fig. 4e). Expression levels of *Tets* varied but the average levels of *Tet1* and *Tet2* seemed not to differ between ESCs cultured with and without feeders (Fig. 4e).

Differentially expressed genes (DEGs) were enriched in pathways including LIF-Stat3, PI3K-Akt, MAPK, TGF-beta, Notch and Wnt signaling (Fig. 4f). A number of genes associated with focal adhesion, ECM–receptor interaction and cytokine–cytokine receptor interaction were upregulated in ESCs with feeders, suggesting more active intercellular interactions. Some cytokines (e.g., ILs and IFNs) and receptors (e.g., ILRs) involved in LIF pathway were up-regulated in feeder + ESCs compared with feeder-free ESCs, while most of LIF targeting genes were unchanged (Supplementary Fig. 4a). Cytokines, ECMs, some growth factors and their corresponding receptors upstream PI3K-Akt were activated in ESCs with feeders (Supplementary Fig. 4b). In Wnt pathway, both activating factors (e.g., Wnts) and inactivating ones (e.g., PEDF and Dkk) were upregulated in feeder + ESCs compared with feeder-free ESCs (Supplementary Fig. 4c). Some ligands (Delta, initiating Notch; Serrate, inhibiting Notch) and receptors were expressed at higher levels in feeder + ESCs than in feeder-free ESCS. Notch targeting genes, *Hes1* and *Hes5* were upregulated in feeder + ESCs (Supplementary Fig. 4d). In Mek-Erk pathway, growth factors (e.g., EGFs and FGFs) and Ras were upregulated, but several Dual Specificity Phosphatases (DUSP1, DUSP9, DUSP10) or MKPs that dephosphorylate and inactivate ERKs^33^, also were upregulated in ESCs with feeders (Supplementary Fig. 4e). p-Erk1/2 level elevated in ESCs cultured with feeders relative to those without feeders, and comparatively was lower than that of MEFs (Supplementary Fig. 4f). Appropriate levels of p-Erk coincided with the high proliferation of ESCs cultured on the feeders. Interestingly, BMP4-Smad pathway and known BMP-Smad targeting genes, *Id2*, *Id3*, *Id4*, were upregulated in ESCs with feeders (Fig. 4g; Supplementary Fig. 4g), suggesting that the activity of BMP-Smad pathway was higher in ESCs cultured with than without feeders.

### BMP4 and Fstl1 secreted by feeders increase *Zscan4*

Furthermore, we attempted to understand signal transduction potentially involving *Zscan4* activation regulated by the feeders at post-transcriptional or translational levels. *Zscan4* was increased in trans-well assay or in ESCs cultured in feeder-conditioned medium (Supplementary Fig. 5a–d), implying that secretory factors may partly activate *Zscan4*. Feeders contribute to ESC self-renewal by secretion of LIF^34^. Under feeder-free culture conditions, increasing concentration of mouse LIF from 1000 U/ml (1×), 2000 U/ml (2×) to even 3000 U/ml (3×) failed to increase *Zscan4* expression in ESCs (Supplementary Fig. 5e). PI3K-Akt and Wnt-catenin pathways also were analyzed. Expression levels of both *Nanog* and *Zscan4* were notably decreased after inhibition of PI3K-Akt pathway in ESCs cultured on feeders (Supplementary Fig. 5f), consistent with the finding on role of PI3K-Akt in regulating *Zscan4* expression^35^. Yet, the active forms of Akt, phosphorylated Akt at Thr308 and Ser473, were downregulated in ESCs cultured on feeders (Supplementary Fig. 5g, h). Although expression of Wnt targeting genes, *Cdx1* and *Axin2*, was slightly increased in ESCs with feeders, both global and nuclear levels of β-catenin protein levels did not differ between −F and +F ESCs (Supplementary Fig. 5i–k). GSK3β inhibitor (CHIR99021, CHIR) that stabilizes β-catenin and enhances Wnt signaling^23^, slightly reduced *Zscan4* expression (Supplementary Fig. 5l). These results suggest that feeders activate *Zscan4* unlikely through LIF, PI3K-Akt, or Wnt-catenin pathways.

*Jagged1* in Notch signaling was the predominant ligand expressed by feeders and *Notch4* highly expressed in ESCs (Supplementary Fig. 6a, b). However, Jagged1-activation peptide did not alter expression of *Zscan4* in two ESC lines cultured without feeders (Supplementary Fig. 6c). Moreover, knockdown of *Notch4* upregulated *Zscan4* in most clones (Supplementary Fig. 6d–f), suggesting that Notch signaling may negatively regulate *Zscan4*^*+*^ state.

**Fig. 5:**
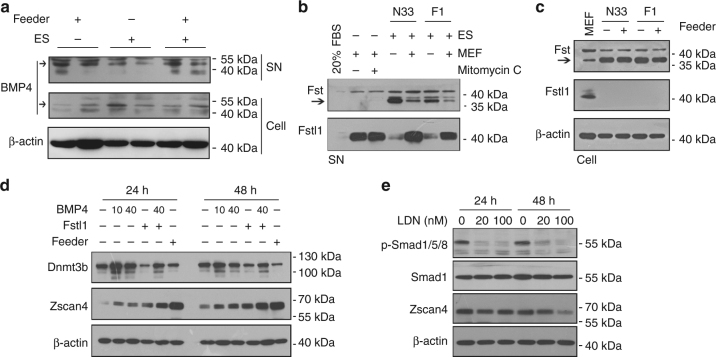
BMP4 and Fstl1 secreted by feeders increase *Zscan4* expression. **a** Western blot analysis of BMP4 in cell extracts (Cell) and in the medium (supernatant, SN) under indicated conditions. **b** Western blot analysis of secreted Fst and Fstl1 by MEF (with or without mitomycin C treatment), ESCs and ESCs + MEF. The band of Fst is indicated by arrow. **c** Western blot analysis of global protein levels of Fst and Fstl1 in MEFs and ESCs. **d** Western blot analysis of Zscan4 and Dnmt3b protein levels after treatment with BMP4 or/and Fstl1. BMP4, 10 ng/ml and 40 ng/ml as indicated; Fstl1, 100 ng/ml. **e** Western blot analysis of pSmad1/5/8 and Zscan4 protein level following treatment with BMP signaling inhibitor LDN-193189 for 24 and 48 h at the indicated concentrations. β-actin served as loading control

BMP4, which acts in combination with LIF to sustain self-renewal of ESCs^36^, was produced by both ESCs and feeders (Fig. 5a). Follistatin (Fst) and Follistatin-like 1 (Fstl1) are important components involved in BMP-Smad pathways^37^. Surprisingly, their secretion patterns were complementary in ESCs and MEFs. ESCs secreted Fst at higher levels but Fstl1 at lower levels than did MEFs, whereas MEFs produced Fstl1 at remarkably higher levels and minimal Fst regardless of mitomycin C treatment (Fig. 5b). Overall expression levels of Fst and Fstl1 were consistent with their secreted quantity (Fig. 5c). Addition of BMP4 or Fstl1 enhanced Zscan4 protein levels to some extent, and their combinations resulted in cumulative effects in feeder-free cultures (Fig. 5d). On the contrary, BMP inhibitor LDN-193189^38^, inhibited phosphorylation of Smad1/5/8 at the concentrations of 20 and 100 nM, slightly reduced expression of Zscan4 24 h following treatment, but evidently reduced Zscan4 by 48 h particularly at 100 nM (Fig. 5e). Together, BMP4 and Fstl1 produced by feeders and activation of BMP-Smad pathway can be partly implicated in facilitating *Zscan4* expression in ESCs.

### Feeders reduce *Dnmt3b* in ESCs

Given the downregulation of genes involved in de novo DNA methylation in ESCs with feeders (Fig. 4a, e), we also tested whether DNA methylation can influence *Zscan4* expression. qPCR and western blot validated that expression levels of Dnmt3a and particularly Dnmt3b were noticeably decreased in ESCs cultured on feeders compared to those without feeders (Supplementary Fig. 7a–c). By ChIP-qPCR analysis, binding of Dnmt3b to *Zscan4* promoter and subtelomeres of chromosomes 7 and 13 was not significantly changed with or without feeders (Supplementary Fig. 7d). DNA methylation levels did not differ between ESCs cultured with and without feeders either, as analyzed by bisulfite sequencing using primers specific for subtelomeres of chromosomes 7 and 13 (Supplementary Fig. 7e). MeDIP (methylated DNA immunoprecipitation)-qPCR analysis confirmed that 5mC levels did not differ at subtelomeric sites and *Zscan4* promoter in ESCs cultured with and without feeders (Supplementary Fig. 7f). These data suggest that DNA methylation levels at subtelomeres or *Zscan4* promoter region may not regulate Zscan4 expression, despite that the feeders reduce *Dnmt3b*.

To test whether reduction of *Dnmt3b* could activate *Zscan4*, we depleted *Dnmt3b* in mouse ESCs by shRNA using two different sequences (KD-1 and KD-2). Dnmt3b was dramatically downregulated, and yet the mRNA and protein levels of Zscan4 varied and showed slight increase in some lines/clones after stable knockdown of *Dnmt3b* for 9 passages (Supplementary Fig. 7g–i), although short-term reduction of Dnmt3b can upregulate Zscan4 at various levels^39^. These data suggest that increased expression of *Zscan4* might not result from repression of *Dnmt3b* in ESCs cultured on feeders.

To determine whether Zscan4 could downregulate Dnmt3b, we sorted Zscan4^+^ and Zscan4^−^ cells by flow cytometry using a *Zscan4* promoter-EGFP ESC line^39^. As expected, Zscan4^*+*^ ES cells expressed Zscan4 mRNA and protein at high levels (~40-folds higher than those of Zscan4^−^ ES cells, Supplementary Fig. 7j, k). In contrast, Zscan4^+^ ESCs expressed Dnmt3b mRNA and protein at lower levels than did Zscan4^−^ ESCs (Supplementary Fig. 7j, k). Zscan4^*+*^ cells exhibited longer telomeres than did Zscan4^−^ cells (Supplementary Fig. 7l-n). Overexpression of *Zscan4* reduced Dnmt3b protein levels (Supplementary Fig. 7o), in agreement with the recent finding that Zscan4 activation of ESCs drastically reduces Dnmts and particularly Dnmt3b levels and results in transient genome-wide DNA demethylation^40^. Moreover, overexpression of *Zscan4* in ESCs culture without feeders noticeably elongated telomeres (Fig. 3f–h).

### 2i suppress *Zscan4* and SSEA1

Interestingly, ESCs cultured in 2i condition undergo global DNA hypomethylation, and particularly Dnmt3a/3b are suppressed by 2i^41,42^. We validated in our ESC lines cultured on feeders that Dnmt3a and Dnmt3b were indeed repressed after 2i treatment (Supplementary Fig. 8a–h). Moreover, 5mC immunofluorescence was decreased in 2i condition (Supplementary Fig. 8f). Without feeders, 2i also suppressed both Dnmt3a/3b and more evidently Dnmt3a (Supplementary Fig. 8i).

**Fig. 6:**
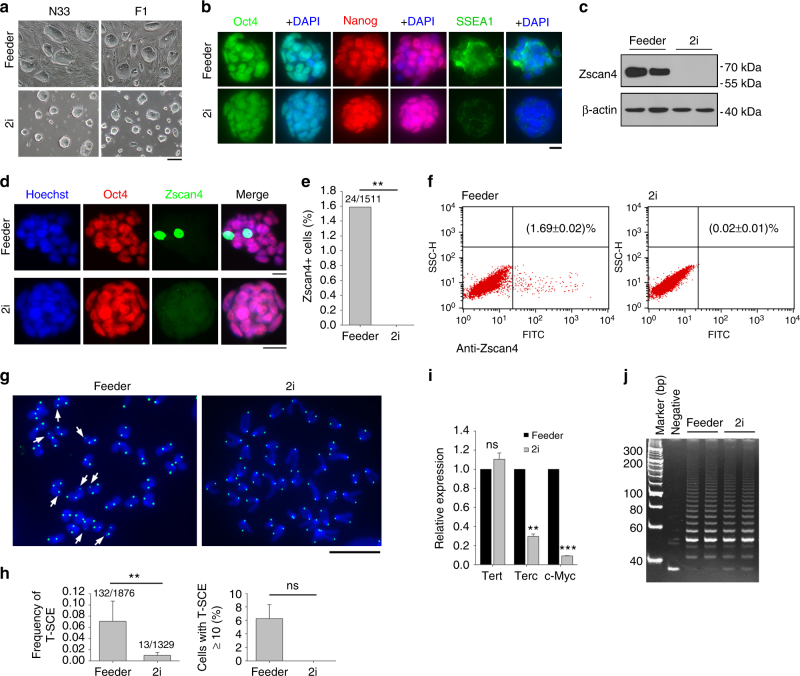
2i repress Zscan4 and SSEA1. **a** Morphology under bright field with phase-contrast optics of N33 and F1 ESCs cultured in serum + feeder + LIF (feeder) or N2B27 + 2i + LIF (2i) conditions. Scale bar, 100 μm. **b** Immunofluorescence of pluripotency-associated markers Oct4, Nanog and SSEA1 in N33 ESCs (five passages). Scale bar, 10 μm. **c** Zscan4 protein levels by western blot (three passages). **d** Immunofluorescence showing co-staining of Oct4 and Zscan4 in N33 ESCs. Scale bar, 10 μm. **e** Percentage of Zscan4^+^ cells in ~1500 ESCs counted. **f** Flow cytometry quadrantal diagram indicating proportion of Zscan4^+^ cells in a ESC population. **g** Representative CO-FISH images showing T-SCE (indicated by white arrows) in feeder- and 2i- ESCs. Scale bar, 10 μm. **h** Frequency of T-SCE (total T-SCE/total chromosomes) and percentage of ESCs with T-SCE foci ≥ 10. **i** Expression of telomerase-related genes *Tert*, *Terc*, and *c-Myc* in feeder- and 2i- ESCs. **j** Telomerase activity measured by TRAP assay. Lysis buffer served as negative control. **P < 0.01, ***P < 0.001, ns not significant (*P* > 0.05). Student’s *t*-test for **i**, and *χ*^2^ test for **e** and **h**

ESCs cultured under either feeder or 2i conditions formed nice tight ESC colonies (Fig. 6a). ESCs cultured in 2i medium without feeders expressed homogeneous Nanog, unlike heterogeneous expression of Nanog in ESCs cultured on feeders (Fig. 6b; Supplementary Fig. 8j, k). SSEA-1 is one of cell surface markers to characterize pluripotent mouse ESCs^43^. Surprisingly, expression of SSEA-1 was reduced in ESCs cultured under 2i condition (Fig. 6b; Supplementary Fig. 8j).

Unlike those of ESCs cultures on feeders, Zscan4 protein levels and proportion of Zscan4^+^ cells were minimal in ESCs cultured under 2i condition without feeders as determined by qPCR (Supplementary Fig. 9a-c), Western blot (Fig. 6c and Supplementary Fig. 9d), fluorescence microscopy (Fig. 6d, e; Supplementary Fig. 8l), and flow cytometry (Fig. 6f and Supplementary Fig. 9e–g). Previous RNA-seq analysis showed that *Zscan4* was not found in 160 genes specifically expressed in ESCs culture under 2i condition (FPKM > 0.5), but in 461 genes expressed only in serum culture^25^. Thus, *Zscan4* is suppressed in ESCs cultured under 2i condition without feeders. However, ESCs cultured on feeders exhibited variable expression levels of Zscan4 after addition of 2i (Supplementary Fig. 8g, h; Supplementary Fig. 9a).

Frequency of T-SCE was reduced in ESCs cultured in 2i medium (Fig. 6g, h). Particularly, ESCs with high T-SCE rate (T-SCE ≥ 10 foci/cell) were exclusively found under feeder culture, in contrast to 2i condition (Fig. 6g, h). Relative expressions levels of *Terc* and *c-Myc* decreased in 2i condition (Fig. 6i), and yet telomerase activity did not differ between ESCs cultured under 2i and feeder conditions (Fig. 6j). Perhaps the template *Terc* levels are sufficient for telomerase activity.

**Fig. 7:**
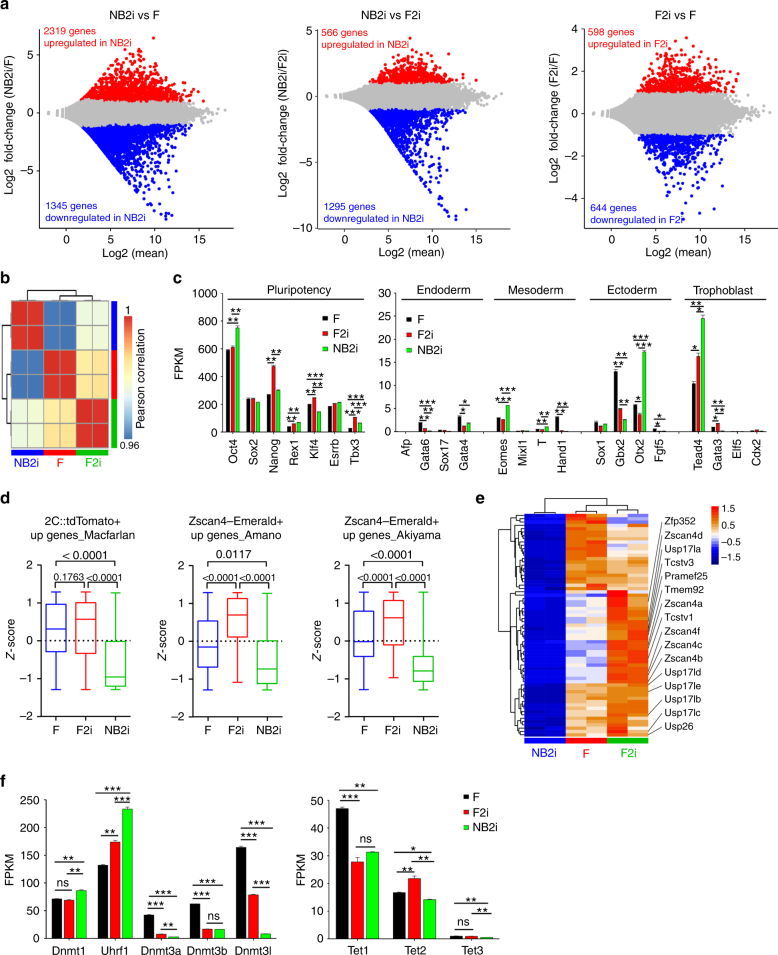
Global gene expression profile in ESCs cultured with feeders and/or 2i. **a** Pairwise comparisons (*P*adj < 0.05, ≥2-fold expression levels) of ESCs cultured in N2B27 + 2i (NB2i) vs serum + feeder (F), NB2i vs serum + feeder (F2i) and F2i vs F to reveal non-redundant, significant changes in gene expression. Red dots represent significantly upregulated genes, blue dots downregulated genes, and gray dots represent genes that are not significantly changed. **b** The Pearson’s correlation coefficient to estimate the relationships between samples at the whole transcriptome level. **c** Expression levels (FPKMs) of pluripotency and lineage marker genes in ESCs under three culture conditions. **d** Z-scores of several lists of 2C genes. Lists used here are based on those of Fig. 4c. **e** Heatmap showing expression of representative 2C genes. **f** Expression levels (FPKMs) of multiple genes involved in DNA (de)methylation in ESCs under three culture conditions. *P < 0.05, **P < 0.01, ***P < 0.001, ns not significant (*P* > 0.05). ANOVA with Fisher’s protected least-significant difference (PLSD) was used for the statistical analysis

RNA-seq analysis revealed differential global transcription profile of ESCs cultured under three conditions, LIF + serum + feeder (F), LIF + serum + feeder + 2i (F2i) and LIF + N2B27 + 2i (NB2i) (Fig. 7a, *P*adj < 0.05, fold change ≥ 2). By Pearson’s correlation, F2i was more similar to F than to NB2i at the transcriptome level (Fig. 7b). Expression levels of pluripotency genes varied among ESCs cultured in F, F2i, and NB2i condition (Fig. 7c; Supplementary Fig. 10a). Representative lineage marker genes generally were repressed under the three culture conditions. While *Gbx2* was expressed at relatively higher level in ESCs cultured with feeder only, *Eomes*, *Otx2*, and *Tead4* expressed at higher levels in NB2i than in F or F2i (Fig. 7c).

Compared with those of NB2i cultures, 2C genes, as described in Fig. 4c, were consistently increased in ESCs under feeder condition and further upregulated under F2i condition (Fig. 7d). Representative 2C genes were enriched in ESCs with F and F2i (Fig. 7e). 2i robustly repressed *Dnmt3a/3b/3l* regardless of feeders (Fig. 7f). Together, the three culture conditions result in distinct transcriptome patterns. Feeder cells facilitate and 2i suppress 2C genes.

### Telomere maintenance of mESCs in 2i culture

Shorter telomeres were found in N33 ESCs cultured in 2i medium without feeders, compared with those of ESCs cultured with feeders or feeders added with 2i at early passage (P5) (Supplementary Fig. 10b, c). Meanwhile, chromosome fusion was exclusively observed in ESCs cultured in 2i condition without feeders, despite no statistical differences on average (Supplementary Fig. 10b, d). Telomere lengths were not evidently changed after 2i treatment in serum + feeder condition (Supplementary Fig. 10b, c).

**Fig. 8:**
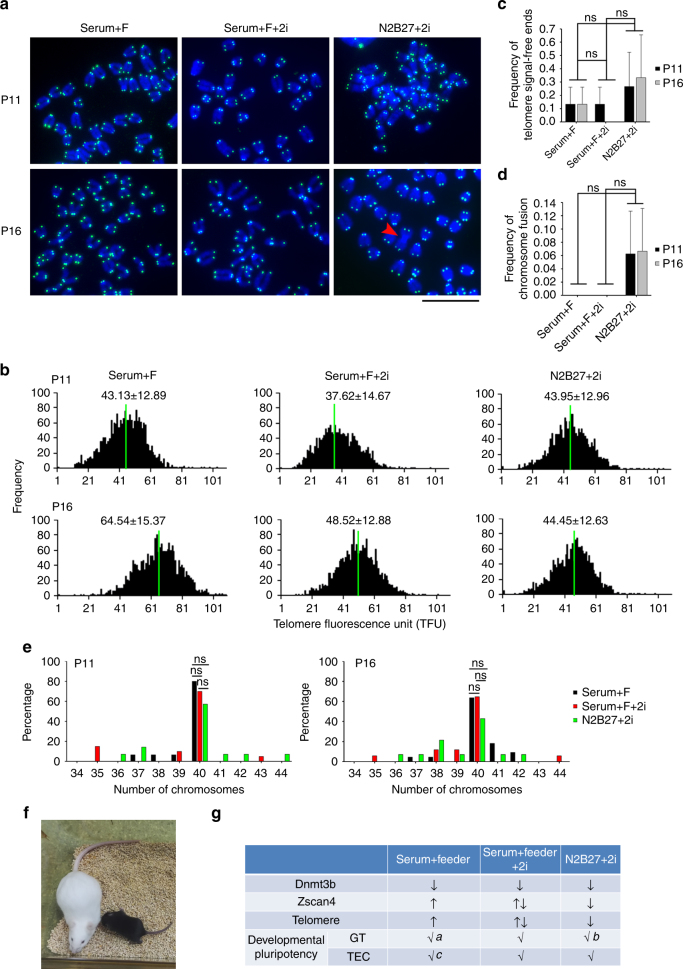
2i fail to maintain telomeres in ESC culture without feeders. **a** Representative telomere Q-FISH images of OG4 ESCs cultured under three culture conditions. Blue, chromosomes stained by DAPI; green dots, telomeres. Red arrowhead indicates chromosome fusion. Scale bar, 10 μm. **b** Histogram displaying distribution of relative telomere length shown as TFU. Green line indicates medium telomere length. Mean ± s.d. of telomere length is shown above each panel. At P16, *P* < 0.0001 (serum + F vs serum + F + 2i; serum + F + 2i vs N2B27 + 2i; serum + F vs N2B27 + 2i). Wilcoxon–Mann–Whitney rank sum test. **c**, **d** Frequency of telomere signal-free ends (**c**) and chromosomal fusion (**d**). ns not significant (*P* > 0.05), ANOVA with PLSD was used for the statistical analysis. **e** Distribution of chromosome numbers in ESCs cultured under three conditions. *n* = 30 spread counted for each group. ns not significant (*P* > 0.05). *χ*^2^ test. **f** Pup (black coat) born from OG4 ESCs cultured in N2B27 + 2i/L condition for four passages by TEC assay. **g** Comparison of expression levels of Dnmt3b and Zscan4, telomere lengths and developmental pluripotency of ESCs cultured under the three culture conditions (all added with LIF). GT germline transmission, TEC tetraploid embryo complementation, ↑ increased, ↓ decreased, ↑↓ vary in different cell lines, √ achieved, a, Huang et al.^19^^,^^61^; b, Ying et al.^23^; c, Chen et al.^60^

Furthermore, we compared telomere lengths of OG4 mESCs in hybrid background by culture for longer-term with feeders or 2i conditions. The OG4 ESC line was newly established from Oct4-ΔPE-GFP mice (see Methods). Telomere lengths of OG4 ESCs were comparable between serum + feeder and 2i conditions, but shorter in serum + feeder + 2i condition by passage 11 (Fig. 8a, b). Notably, however, ESCs cultured in serum + feeder and serum + feeder + 2i conditions exhibited telomere elongation with increasing passages (to P16), compared with that of passage 11, whereas ESCs cultured in 2i condition without feeders did not undergo telomere elongation during passages (Fig. 8a, b), indicating impaired telomere extension of ESCs cultured in 2i condition without feeders during extended culture. Telomeres of ESCs at P16 were longer in serum + feeder condition than in serum + feeder + 2i or 2i condition. Moreover, chromosome fusion was only observed in ESCs cultured in 2i condition without feeders, despite without statistical differences on average (Fig. 8a, d). Further, ESCs cultured under three conditions maintained relatively normal karyotypes at passage 11, but the number of cells with normal ploidy (40 chromosomes) was reduced in 2i condition compared with feeder and feeder + 2i conditions (57.1% in 2i, 80.0% in feeder and 70.0% in feeder + 2i condition). Feeder and feeder + 2i ESCs sustain normal karyotypes with more than 60%, whereas only 42.3% ESCs cultured in 2i showed normal ploidy at passage 16 (Fig. 8e), indicating that sustained 2i condition may lead to aneuploidy.

To test the developmental pluripotency of ESCs cultured under 2i conditions, we performed TEC assay. OG4 ESCs cultured in 2i medium for additional 4-5 passages (total P9-10) were able to give rise to full-term development shown as complete-ESC pups by TEC assay. The same ESC line also could generate complete-ESC pups after long-term culture (to P15–16) at lower efficiency, but these ESC pups failed to survive to the adulthood (Fig. 8f, Table 1). ESCs at P9-10 maintained in serum + feeder + 2i condition also could generate live-born pups, although the pups could not live to adults, and these ESCs after extended cultures (P15) failed to give rise to live-born pups (Table 1). Hence, 2i culture condition confers developmental pluripotency by TEC assay, but long-term culture under 2i condition could negatively affect survival of the ESC pups.

**Table 1:** Summary of TEC assay

Culture condition	Passage no.	No. of embryos transferred	Pups born (full-term)	Breathing	Adults
Serum + feeder + 2i	P9-10	79	2	2	0
	P15	39	0	0	0
N2B27 + 2i	P9-10	64	8	7	2^a^
	P15-16	69	2	2	0

Full-term, mice were fully developed at the time of birth. Breathing, mice were able to breath on their own after birth. Adults, mice that lived longer than 5 weeks^44^

^a^One died 37 days after birth, the other one, now over 10 months old and appears healthy

## Discussion

We show that telomeres are longer in ESCs cultured with feeders than those without feeders, important for chromosomal stability and unlimited self-renewal of ESCs. ESCs cultured with feeders or without feeders or those in 2i conditions express similarly high telomerase activity. Hence telomerase activity itself may not explain the differential telomere lengths observed in these culture conditions. Nevertheless, feeders increase Zscan4 expression and decrease Dnmt3b, and promote telomere elongation, maintaining normal ploidy. 2i added in feeder culture condition results in consistent repression of Dnmt3a/3b, variable expression of Zscan4, and relatively stable telomere lengths. N2B27 + 2i/L condition suppresses Dnmt3a/3b as well as Zscan4, and can cause shorter telomeres and chromosome fusion in ESCs (Fig. 8g). The defective adult development of complete-ESC pups might be related to telomere defects such as shortest telomeres and chromosome fusion of the ESCs after extended culture. In addition to chromosomal aberrations, irreversible erasure of genomic imprints resulting from downregulation of DNA methyltransferases in prolonged culture of ESCs in 2i/L condition also could contribute to impaired developmental potential and failed survival to adulthood of complete-ESC pups if any^44,45^.

We propose that periodic production and secretion of specific factors by feeders e.g. Fstl1, and others to be identified, and in close cell contact with feeders activate sporadic expression of 2C-genes and critically Zscan4 to lengthen telomeres. Zscan4 is activated in mESCs with longer cell cycle phase when telomeres are short^46^, or activated in response to artificial telomere shortening by depletion of telomerase^19^. Contrary to the expectations, Zscan4^+^ cells are not associated with high developmental potency compared with Zscan4^-^ cells^31^. Too frequent activation of Zscan4, resulting in more Zscan4^+^ cells in culture, actually lower the average potency of ESCs^31^. Our data also validate the highly dynamic nature of Zscan4 expression and degradation in heterogeneous ESCs. Frequency of T-SCE overall is low in a ESC population. However, T-SCEs at high frequency (≥10 foci/cell) exclusively emerge in ESCs cultured with feeders, associated with telomere elongation, in contrast to those without feeders or in 2i culture condition (Fig. 2j, k and 6g, h), linked to shorter telomeres and chromosome fusion with increasing passages.

Interestingly, transcription factor, e.g., Nanog heterogeneity also provides a stochastic advantage in pluripotent stem cells^8^. Suppression of the heterogeneity, e.g., by 2i culture without feeders, not only represses Dnmt3a/3b, leading to hypomethylation, but also reduces Nanog heterogeneity and abolishes transient *Zscan4* expression, which can contribute to telomere shortening following long-term passages of ESCs. Similarly, the proportion of MERVL^+^Zscan4^+^ cells and relative expression levels of Zscan4 are notably reduced upon long-term naive 2i culture of E14 ESCs derived from 129/Ola^40^ or the 2 CːːtdTomato reporter ESC lines^10^. Overexpression of *Zscan4* can reduce Dnmt3a/b levels. Sustained global hypomethylation and inhibition of periodic expression of *Zscan4* resulting from prolonged culture in 2i condition and without feeders could reduce telomere maintenance and chromosomal stability of ESCs.

We have shown a novel mode of action on telomeres of MEF feeders to maintain long-term stable self-renewal of mESCs. We noticed that the average telomere length is only relatively longer in ESCs cultured with feeders than those without feeders. Nevertheless, telomere signal-free ends indicative of shortest telomeres are consistently found in ESC culture without feeders, despite at low frequency, unlike ESCs cultured with feeders. The biological significance of these mild telomere defects warrants further investigation. Presumably, the critically short telomere might contribute to chromosome instability in ESCs. This is supported by the original findings that it is the shortest telomere, not the average telomere length, that drives chromosome instability, affecting cell viability and transformation^47,48^. Moreover, short telomeres themselves, rather than lack of telomerase activity, impair stem cell function^49^. Short telomeres in ESCs lead to unstable differentiation^50^ and decreased developmental pluripotency^19^.

The genetic background may influence the reliance of feeders on telomere maintenance and self-renewal of ESCs. The popular inbred strain of mES cell lines from 129 backgrounds confers high ability to receive the LIF signal to sustain self-renewal and prevent differentiation^51^. The 129-derived ESCs are able to continue self-renewal without feeder cells in serum + LIF culture condition with keeping the high ability to contribute to chimeric embryos at E13.5^52^. In our study, N33, AG-B3, AG-J2 ESCs were derived from C57BL/6J, OG4 from C57BL/6×CBA and F1 from B6C3F1. C57BL/6J-derived ESCs might be less stable in serum + LIF without feeders than 129-derived ESCs. Nevertheless, these various ESC lines showed telomere elongation and sporadically higher Zscan4 expression by culture with feeders than without feeders. The 129-derived ESCs periodically express Zscan4 in both serum + LIF and 2i + LIF culture conditions without feeder cells during short experimental period^46^. Similarly, J1 ESCs originally derived from 129S4/SvJae strain also express Zscan4 without feeders, but display longer telomeres and higher Zscan4 expression by culture with the feeders (Supplementary Fig. 2). The passage number of the J1 ESCs was unknown and presumably could be pretty high. The J1 ESCs without feeders acquired sporadic expression of Zscan4, but the expression level of Zscan4 by immunofluorescence intensity in the Zscan4^+^ cells was lower than that of N33 ESCs cultured on the feeders (Supplementary Fig. 2e; Fig. 2h). In relevance, the J1 ESCs exhibited high incidence of aneuploidy and failed to generate germline chimeras.

Previous study demonstrated that V6.5 ESCs derived from C57BL/6×129/SvF1 retained the ability to produce tetraploid chimeras after prolonged passage (18–19) in serum + LIF without feeders^31^. Using ESC lines in 129×C57 genetic background, we also find that ESCs in the presence of feeders sporadically express Zscan4 at higher levels and exhibit longer telomeres than those without feeders, although telomeres shorten to less extent compared with those of C57 genetic background cultured without feeders (Supplementary Fig. 11). Chromosome fusions are not observed in 129×C57 ESCs cultured without feeders for 11 passages (Supplementary Fig. 11), suggesting that ESCs at 129×C57 background might be less sensitive to feeder-free cultures. However, Amano *et al*. also provided strong evidence that the ability to generate chimeras of the ESCs without feeders is markedly reduced with increasing passages. Furthermore, reduced expression level of *Zscan4* is correlated with impaired developmental pluripotency and forced expression of *Zscan4* indeed can maintain telomere length and normal karyotypes and enhance developmental pluripotency^31^. Notably, no normal chromosome karyotypes are found in V6.5 ESCs at P30, in contrast to V6.5 ESCs with forced expression of *Zscan4*, showing normal ploidy. It remains unclear whether the TEC embryos and newborn can survive to adulthood. Our data shows that TEC pups also could be produced from ESCs cultured in 2i/L condition for more than 10 passages (Fig. 8), but they hardly survived to adulthood, coincident with the recent findings^44^. Together, sufficient telomere maintenance and chromosomal stability by either forced expression of *Zscan4* or by feeders-facilitated periodic expression of *Zscan4* are important for developmental pluripotency and long-term stable self-renewal of mESCs, and survival to adulthood of ESC pups.

Aneuploidy and genomic instability have been found to accumulate during prolonged culture of human pluripotent stem cells^53,54^, but it remains to be determined whether feeder-free cultures lead to increased incidence of chromosomal abnormality. Moreover, feeder cells also have been used in achieving human naive-like pluripotent stem cells^55–58^, and extended pluripotent stem (EPS) cells in both mice and humans^59^. Our data highlight a critical role of feeders in telomere maintenance of naive mouse ESCs and warrant careful scrutiny of telomeres and genomic stability of ESCs cultured for long-term under feeder-free conditions. On the other hand, human telomere length, typically 5–15 kb^30^, is much shorter than mouse telomeres. Human ESCs also have much shorter telomeres than do mouse ESCs, and are more sensitive to telomere shortening^20^. Further understanding of the role and mechanisms of feeder cells in maintaining telomeres may have relevance to potential clinical application of human ESCs.

## Methods

### Mice and ES Cell culture

Mice were housed and cared in the College Animal Facility and the use of mice for the research approved by Institutional Animal Care and Use Committee at Nankai University. All the animal experiments were performed following the ethical guidelines approved by Tianjin Animal Management Committee. Following mouse ES cell lines were used in this study. N33 ESC line was derived from C57BL/6J mice^19^, F1 ESC line from B6C3F1 mice^60^, and NF2 ESC cell line from Nanog-EGFP transgenic mice^61^. J1 ESCs cultured without feeders were 129S4/SvJae origin. AG-B3 and AG-J2 ESC lines were derived in our laboratory from actin-GFP mice in C57BL/6J origin, and exhibited germline competence in the resultant chimeras. OG4 ESC line expressing distal Oct4-GFP was recently established from Oct4-GFP mice in C57BL/6J×CBA origin and characterized based on the method described^61^, but with addition of 2i in the medium. Oct4-GFP (OG) mice that carry Oct4 distal promoter-driven GFP were purchased from Model Animal Research Center of Nanjing University. The transgenic GFP expression of the reporter is under the control of Oct4 promoter and distal enhancer, but the proximal enhancer region is deleted, so the Oct4-ΔPE-GFP transgenic mice have been used for establishing naive mouse ESCs^62–64^. The OG4 ESC lines passed the germline chimera test.

New ESC lines from 129×C57/B6 genetic background also were derived as previously described^19,60^. Briefly, intact blastocysts from 129S6 (♂) × C57/B6 (♀) mice were seeded on feeder layers of mitomycin C-treated MEF cells, prepared on 0.1% gelatin-treated four-well culture dish, in ESC medium consisting of knockout DMEM (GIBCO, Gaithersburg, MD), 20% knockout serum replacement (KSR, Invitrogen), supplemented with 1000 U/ml mouse ESGRO leukemia inhibitory factor (LIF; Chemicon International Inc., Temecula, CA, USA), 1 μM PD0325901, 0.1 mM NEAA, 1 mM l-glutamine, 0.1 mM β-mercaptoethanol, 50 IU/ml penicillin, and 50 IU/ml streptomycin. Half of the medium was changed daily, beginning on the second day after blastocysts were seeded. Approximately 7 days after seeding, ICM outgrowths were mechanically removed and digested with 0.25% trypsin-ETDA (GIBCO) into small clumps, digestion stopped with trypsin inhibitor (Sigma, T6414), and cell suspensions reseeded on fresh feeders. Except for NF2 ESC line which is XX, all other ESC lines are XY.

For routine feeder culture condition, ESCs were cultured under 5% CO_2_ at 37 °C on mitomycin C-treated MEF feeder in ES cell culture medium consisting of knockout DMEM supplemented with 20% fetal bovine serum (FBS, ES quality, Hyclone), 1000 U/ml leukemia inhibitory factor (LIF) (ESGRO, Chemicon), 0.1 mM non-essential amino acids, 0.1 mM β-mercaptoethanol, 1 mM l-glutamine, and penicillin (100 U/ml) and streptomycin (100 µg/ml)^19,65^. For feeder-free culture (serum condition), plates were pre-coated with gelatin or E-cad-fc^29^. ESCs were cultured in ES cell culture medium as above. Chemically defined serum and feeder-free culture conditions (N2B27 + 2i) were based on previous reports^23^. ESCs were cultured on poly-l-ornithine and laminin-coated plates using N2B27 medium (DMEM/F12 and Neurobasal medium mixed at a ratio of 1:1, 1xN2 supplement, 1xB27 supplement, 2 mM l-glutamine, 0.1 mM β-mercaptoethanol, 0.1 mM nonessential amino acids, 100 units/ml penicillin, 100 μg/ml streptomycin) supplemented with Gsk3β inhibitor (CHIR99021, 3 μM) and Mek inhibitor (PD0325901, 1 μM) and 1,000 U/ml LIF.

### Chimera and tetraploid embryo complementation assay

ESCs were injected into four- or eight-cell embryos as hosts from mice with different genetic background using a Piezo injector^19,61^. Injected embryos were cultured overnight in KSOM_AA_ medium. Blastocysts were transferred into uterine horns of 2.5 dpc pseudo-pregnant Albino Kunming mice. Chimeras were identified initially by coat color. The contribution of ESCs to various tissues in chimeras was confirmed by standard DNA microsatellite genotyping analysis using D12Mit136 primers: 5’-TTT AAT TTT GAG TGG GTT TGG C-3’ and 5’-TTG CTA CAT GTA CAC TGA TCT CCA-3’. Chimeras were mated with albino strain ICR mice to further examine their germline transmission competence.

For tetraploid embryo complementation (TEC) assay, tetraploid (4 N) embryos were produced by the electrofusion of 2-cell embryos collected from Balb/c mice. About 10–15 ESCs were injected into one tetraploid blastocyst and the injected blastocysts transferred into surrogate Kunming females.

### Telomere quantitative fluorescence in situ hybridization

Telomere length and function (telomere integrity and chromosome stability) were estimated by telomere Q-FISH^19^. Cells were incubated with 0.3 μg/ml nocodazole for 3 h to enrich cells at metaphases. Metaphase-enriched cells were exposed to hypotonic treatment with 75 mM KCl solution, fixed with methanol: glacial acetic acid (3:1), and spread onto clean slides. Telomeres were denatured at 80 °C and hybridized with FITC-labeled (CCCTAA)_3_ peptide nucleic acid (PNA) telomere probe (0.5 μg/ml) (Panagene, Korea). Chromosomes were stained with 0.5 μg/ml DAPI. Fluorescence from chromosomes and telomeres was digitally imaged on a Zeiss Axio Imager Z2 with FITC/DAPI filters, using AxioCam and AxioVision software 4.6. Telomere length shown as telomere fluorescence intensity was integrated using the TFL-TELO program (a gift kindly provided by P. Lansdorp).

### Chromosome orientation fluorescence in situ hybridization

CO-FISH assay was performed based on the method described^66^, with minor modification. Subconfluent ES cells were incubated with BrdU (10 μM) for 10-12 h. Nocodazole was added to enrich metaphase 2 h prior to cell harvest, and metaphase spreads were prepared as described above for telomere QFISH. Chromosome slides were treated with RNaseA, fixed with 4% formaldehyde, then stained with Hoechst 33258 (0.5 mg/ml) for 15 min and exposed to 365 nm UV light for 40 min. The BrdU-substituted DNA was digested with Exonuclease III (Takara). The slides were then dehydrated through ethanol series and air-dried. PNA-FISH was performed with FITC-OO-(CCCTAA)_3_ (Panagene, F1009). Slides were hybridized, washed, dehydrated, and counterstained with VectaShield antifade medium (Vector), containing 1.25 μg/ml DAPI. Digital images were captured using a CCD camera on a Zeiss Axio-Imager Z1 microscope.

### Telomere restriction fragment analysis

TRF analysis was performed using a commercial kit (TeloTAGGG Telomere Length Assay, catalog no. 12209136001, Roche Life Science). Cells were pretreated with RNaseA and Proteinase K (PCR Grade, 03115879001, Roche Life Science), followed by extraction using phenol: chloroform: isoamyl alcohol, digested with *MboI* (R0147, NEB) at 37 °C overnight and electrophoresed through 1% agarose gels in 0.5 × TBE at 14 °C using a CHEF Mapper pulsed field electrophoresis system (Bio-rad). Auto algorithm was used to separate DNA samples with a size range from 5 to 150 kb. The gel was blotted and probed using reagents in the kit.

### Quantitative real-time PCR

ESCs cultured with feeders were removed off feeders twice based on their differences in the adherence to the bottom of dish. Total RNA was extracted with RNeasy Mini Kit (Qiagen), according to manufacturer’s instructions. Two micrograms of RNA were reversely transcribed into cDNA using MMLV reverse transcriptase (Invitrogen). Real-time quantitative PCR reactions were set up in duplicate with the Faststart Universal SYBR Green Master Mix (Roche) and run on the iQ2 and MyiQ™ Real-Time PCR Detection System (Bio-Rad). GAPDH was set as the internal control. The primers are listed in Supplementary Table 2.

### Telomerase activity by TRAP assay

Telomerase activity was measured by the Stretch PCR method according to the manufacturer’s instruction using TeloChaser Telomerase assay kit (T0001, MD Biotechnology). Briefly, about 2.5 × 10^4^ cells from each sample were lysed. Lysis buffer served as negative controls. PCR products of cell lysates were separated on non-denaturing TBE-based 12% polyacrylamide gel electrophoresis and visualized by ethidium bromide staining.

### Immunofluorescence microscopy

ESCs were grown on gelatin-treated coverslips and fixed with freshly prepared 3.7% paraformaldehyde in PBS, permeabilized in 0.1% Triton X-100 in blocking solution (3% goat serum and 0.1% BSA in PBS) for 30 min, washed three times, and left in blocking solution for 1 h. Samples were incubated overnight at 4 °C with primary antibodies (for antibody details, see Supplementary Table 3), washed three times, and incubated for 2 h with secondary antibodies, Goat Anti-Mouse IgG (H + L) FITC or Goat Anti-Rabbit IgG (H + L) Alexa Fluor 594 (Jackson). Samples were washed and counterstained with 0.5 μg/ml Hoechst 33342 or DAPI in Vectashield mounting medium. Fluorescence was detected and imaged using a Zeiss Imager Z1 microscope or Confocal Laser Scanning Microscope (Zeiss LSM710).

### Immunofluorescence-telomere FISH

ESCs were subject to immunostaining using Zscan4 antibody as described in Immunofluorescence microscopy section. Excessive secondary antibody was washed with PBS, cells were fixed in 4% formaldehyde for 2 min, dehydrated with ethanol, and incubated with Cy3-labeled telomeric PNA probe as described in Q-FISH section. Fluorescence was imaged using Zeiss Imager Z1 fluorescence microscope.

### Flow cytometry analysis

For analysis of endogenous Zscan4 expression profile (percentage of Zscan4^+^ cells and fluorescence intensity), ESCs were collected and washed with cold PBS, then fixed in cold 70% ethanol, permeabilized in 0.1% Triton X-100 in blocking solution (3% goat serum and 0.1% BSA in PBS) for 30 min, washed three times, and left in blocking solution for 1 h. ESCs were incubated overnight at 4 °C with primary antibody against Zscan4 (1:200), washed three times, and incubated for 2 h with secondary antibodies, FITC Goat Anti-Rabbit IgG (554020, BD). Samples were washed three times with PBS and FACS analysis was performed using a FACScan Flow Cytometer (BD Biosciences). FACS analysis of *Zscan4*-EGFP ESCs was carried out as described^39^.

### ChIP-qPCR

ChIP-qPCR analysis was performed based on the method described^67^. Briefly, ESCs were collected after removing off feeders, and fixed with 1% paraformaldehyde, lysed, and sonicated to achieve the majority of DNA fragments with 100-1000 bp. DNA fragments were enriched by immunoprecipitation with 5 μg Dnmt3b antibody and dynabeads M280 (Life Technologies). The immunoprecipitated material was eluted from the beads by heating for 30 min at 68 °C. To reverse the crosslinks, samples were incubated with Proteinase K at 42 °C for 2 h followed by 67 °C for 6 h. The samples were then extracted with phenol: chloroform: isoamyl alcohol (25: 24: 1, pH > 7.8) followed by chloroform, ethanol precipitated in the presence of glycogen, and re-suspended in TE buffer. ChIP enriched DNA was analyzed by qPCR and β-actin locus served as control. Primers for *Zscan4* loci and sub-telomeres are listed in Supplementary Table 4.

### MeDIP-qPCR

MeDIP-qPCR assay was carried out^68^, with minor modifications. Genomic DNA was extracted by overnight Proteinase K treatment, phenol: chloroform: isoamylalcohol extraction and RNaseA digestion. Prior to carrying out MeDIP, genomic DNA was sonicated to fragments ranging from 100 to 1,000 bp. Four microgram of fragmented DNA was used for each assay. DNA were denatured for 10 min at 99 °C and immunoprecipitated for 2 h at 4 °C with 1 μg antibody against 5-methylcytidine (Active Motif, #39649) in a final volume of 500 μl IP buffer (10 mM sodium phosphate (pH 7.0), 140 mM NaCl, 0.05% Triton X-100). Dynabeads with M-280 sheep antibody to mouse were used to perform IP. The beads were treated with Proteinase K for 3 h at 50 °C and DNA recovered by phenol: chloroform: isoamylalcohol extraction.

### DNA methylation analysis

DNA methylation analysis of subtelomeric regions was performed by bisulphite sequencing using EpiTect Bisulfite Kit (QIAGEN). Automatic sequencing of 12 colonies for each sequence was carried out to obtain the methylation status of all subtelomeric CpG islands. Bisulfite genomic-sequencing primers were designed for subtelomeric regions in chromosomes 7 and 13, according to the criteria that they were the first CpG islands found from the ends of the corresponding chromosome, with one subtelomeric region for each chromosome. Primers for bisulfite sequencing are listed in Supplementary Table 5.

### TCA protein precipitation

Secreted proteins were precipitated using trichloroacetic acid (TCA, T6399, Sigma). Cells were washed three times with PBS and transferred to serum-free media for 24 h, and supernatants collected. The supernatants were centrifuged twice and filtered with 0.45 μm Millex-GV Syringe Filter Unit (Millipore) to remove cells completely. One-tenth volume of TCA was added to supernatants, incubated, centrifuged and washed twice with cold acetone. The pellets were dried, dissolved using 1 × SDS buffer, loaded onto SDS-PAGE and analyzed by western blot.

### Western blot

The cells were collected and resuspended in cell lysis buffer containing 50 mM Tris (pH 7.4), 150 mM NaCl, 1 mM EDTA, 1 mM EGTA, 1 mM NaF, 1 mM Na_3_VO_4_, 1% Triton X-100, 10% glycerol, 0.25% deoxycholate, and 0.1% SDS. Lysates were electrophoresed using SDS–PAGE and blotted onto polyvinylidene difluoride (PVDF) membrane. Membranes were blocked with 5% nonfat milk or 5% BSA solution for 2 h. Samples were probed with primary antibodies overnight at 4 °C (for antibody details, see Supplementary Table 3). Secondary antibodies HRP conjugated donkey anti-Rabbit IgG (GE Healthcare NA934V) or goat anti-mouse IgG (H + L) (ZB2305) were diluted at 1:5000. Protein band was visualized using Supersignal West Pico Chemiluminescent Substrate (Millipore). Uncropped scans can be found in Supplementary Fig. 12.

### Knockdown of *Dnmt3b* and *Notch4* by shRNA

*Dnmt3b* shRNA sequences were synthesized and cloned into pSIREN-RetroQ^39^, according to manufacturer’s instructions. Knockdown (KD) was achieved using two different sequences specifically targeting to mRNA of *Dnmt3b*. The mock KD shRNA without sequence homology to mouse genes served as negative control. pSIREN-RetroQ control and KD plasmid (2 μg) were introduced into J1 ESCs using lipofectamine^TM^ transfection reagent (Invitrogen), according to the manufacturer’s recommendation. ESCs were selected with 500 µg/ml G418 for about 1 week, and the stable knockdown clones picked. The *Dnmt3b* KD sequences are listed in Supplementary Table 6. For stable knockdown of *Notch4*, shRNA sequences were synthesized and cloned into pSIREN-RetroQ, according to manufacturer’s instructions. A packaging cell line—Plat-E (5 × 10^5^) were transfected with 2 μg plasmid using lipofectamine^TM^2000. After 48 h, the supernatant of recombinant retrovirus was collected and used to transfected ESCs (2 × 10^5^). The clones were selected by 10 μg/ml puromycin, and survival ones were picked. The *Notch4* KD sequences are listed in Supplementary Table 6.

### Generation of *pZscan4*-EGFP ESCs

p*Zscan4*-EGFP vector was constructed as previously reported^39,65^. Briefly, a putative *Zscan4* promoter containing the 2570 bp upstream sequences from the *Zscan4c* start codon^11^, was amplified from mouse F1 ES cell genomic DNA with TransStar Fastpfu polymerase (Transgen) using the following primers: forward: AGAGATGCTTCTGCATCTGT; reverse: TGTGGTGACAATGGTGTGAAAG. The PCR product was inserted into pEGFP-1 vector at SalI/KpnI sites. The resultant vector was linearized by XhoI digestion. ESCs were transfected with 2 µg linearized vector using Lipofectamine ^TM^2000 (Invitrogen) and selected with 500 µg/ml G418 (Invitrogen) for about 1 week, and clones with bright green fluorescence were picked and expanded for further experiments.

### Generation of *Zscan4* overexpression and knockdown ESCs

The full length *Zscan4c* CDS were cloned into expression vector pCMV-Tag2B. J1 ESCs were transfected with 2 μg linearized pCMV-*Zscan4c* vector or empty vector served as control using Lipofectamine ^TM^2000 (Invitrogen) and then selected with 500 µg/ml G418 for about 1 week. The resistant clones were picked to achieve stable *Zscan4* overexpression or control ESC lines, which were cultured without feeders for further experiments. ESCs were also transfected with pBase-*Zscan4c* for 48 h to confirm reduced Dnmt3b protein levels after *Zscan4* overexpression.

Control and two different shRNA sequences against *Zscan4c* mRNA were used for *Zscan4* knockdown experiments. The sequences were cloned into pSIREN-RetroQ (Clontech) and the resultant vectors and control vectors were introduced into ESCs using Lipofectamine ^TM^2000 (Invitrogen). ESCs were selected with 2 µg/ml puromycin for about 1 week, and the stable knockdown clones were picked and maintained on feeders to perform further analysis. The 19 nuclotide sequences of *Zscan4* shRNA are listed in Supplementary Table 6.

### RNA sequencing and bioinformatics analysis

ESCs were harvested and total RNA extracted using Qiagen RNeasy Mini kit, according to the manufacturers’ instruction, including a DNAse digestion. Quality control of extracted RNA, construction of an RNA-sequencing library and sequencing on BGISEQ-500 was performed at Beijing Genomics Institute (BGI). Briefly, the poly-A containing mRNA molecules were purified using poly-T oligo-attached magnetic beads. The selected RNA was fragmented and copied into first strand cDNA, which was followed by second strand cDNA synthesis. The cDNA fragments were added by a single “A” base and subsequently ligated with the adapter. The products were then purified and enriched with PCR amplification. PCR yield was quantified and samples were pooled together to make a single-strand DNA circle (ssDNA circle), generating the final library. DNA nanoballs (DNBs) were generated with the ssDNA circle by rolling circle replication (RCR) to enlarge the fluorescent signals at the sequencing process. The DNBs were loaded into the patterned nanoarrays and single-end read of 50 bp were read through on the BGISEQ-500 platform for further data analysis.

For bioinformatics analysis, the clean reads were mapped to the *Mus musculus* mm10 reference genome using *Bowtie2*. Reads were assigned and counted to genes using the *RSEM*. The resulting matrix of read counts was then loaded into *RStudio* (*R* version 3.4.2), and *DESeq2* were used to identify differentially expressed genes. Functional enrichment (GO annotation, KEGG) of gene sets with different expression patterns was performed using *clusterProfiler*. The heat maps were drawn by the function “pheatmap” of R packages “*pheatmap*“ and correlation coefficients were calculation by the function “cor” in *R*. Scatter plots were generated using the “*ggplot2*“ package to graphically reveal genes that differ significantly between two samples.

### Statistical analysis

Data were analyzed by ANOVA with Fisher’s protected least-significant difference (PLSD) using the StatView software from SAS Institute Inc. (Cary, NC), two-tailed Student’s *t*-test, *χ*^2^ test or Wilcoxon–Mann–Whitney rank sum test dependent on specific experiments, and the *P* value was calculated. Statistically significance was defined as *P* < 0.05, *P* < 0.01 or *P* < 0.001.

### Data availability

RNA-Seq data have been deposited in the National Center for Biotechnology Information Gene Expression Omnibus (GEO) database (https://www.ncbi.nlm.nih.gov/geo) under the GEO Series accession number GSE 109418.

## Electronic supplementary material

Supplementary Information

## References

[1] Evans MJ, Kaufman MH (1981). Establishment in culture of pluripotential cells from mouse embryos. Nature.

[2] Eggan K (2002). Male and female mice derived from the same embryonic stem cell clone by tetraploid embryo complementation. Nat. Biotechnol..

[3] Nagy A, Rossant J, Nagy R, Abramow-Newerly W, Roder JC (1993). Derivation of completely cell culture-derived mice from early-passage embryonic stem cells. Proc. Natl Acad. Sci. USA.

[4] Jaenisch R, Young R (2008). Stem cells, the molecular circuitry of pluripotency and nuclear reprogramming. Cell.

[5] Thomson JA (1998). Embryonic stem cell lines derived from human blastocysts. Science.

[6] Fang R (2014). Generation of naive induced pluripotent stem cells from rhesus monkey fibroblasts. Cell Stem Cell.

[7] Hayashi K, Lopes SM, Tang F, Surani MA (2008). Dynamic equilibrium and heterogeneity of mouse pluripotent stem cells with distinct functional and epigenetic states. Cell Stem Cell.

[8] Torres-Padilla ME, Chambers I (2014). Transcription factor heterogeneity in pluripotent stem cells: a stochastic advantage. Development.

[9] Kumar RM (2014). Deconstructing transcriptional heterogeneity in pluripotent stem cells. Nature.

[10] Macfarlan TS (2012). Embryonic stem cell potency fluctuates with endogenous retrovirus activity. Nature.

[11] Zalzman M (2010). Zscan4 regulates telomere elongation and genomic stability in ES cells. Nature.

[12] Guo G (2016). Serum-based culture conditions provoke gene expression variability in mouse embryonic stem cells as revealed by single-cell analysis. Cell Rep..

[13] Kolodziejczyk AA (2015). Single cell RNA-sequencing of pluripotent states unlocks modular transcriptional variation. Cell Stem Cell.

[14] Palm W, de Lange T (2008). How shelterin protects mammalian telomeres. Annu. Rev. Genet.

[15] Blackburn EH, Epel ES, Lin J (2015). Human telomere biology: a contributory and interactive factor in aging, disease risks, and protection. Science.

[16] Marion RM (2009). Telomeres acquire embryonic stem cell characteristics in induced pluripotent stem cells. Cell Stem Cell.

[17] Wang F (2012). Molecular insights into the heterogeneity of telomere reprogramming in induced pluripotent stem cells. Cell Res..

[18] Liu Y (2000). The telomerase reverse transcriptase is limiting and necessary for telomerase function in vivo. Curr. Biol..

[19] Huang J (2011). Association of telomere length with authentic pluripotency of ES/iPS cells. Cell Res..

[20] Yang C (2008). A key role for telomerase reverse transcriptase unit in modulating human embryonic stem cell proliferation, cell cycle dynamics, and in vitro differentiation. Stem Cells.

[21] Liu L (2017). Linking telomere regulation to stem cell pluripotency. Trends Genet..

[22] Xu C (2001). Feeder-free growth of undifferentiated human embryonic stem cells. Nat. Biotechnol..

[23] Ying QL (2008). The ground state of embryonic stem cell self-renewal. Nature.

[24] Buehr M (2008). Capture of authentic embryonic stem cells from rat blastocysts. Cell.

[25] Marks H (2012). The transcriptional and epigenomic foundations of ground state pluripotency. Cell.

[26] Singer ZS (2014). Dynamic heterogeneity and DNA methylation in embryonic stem cells. Mol. Cell.

[27] Li M, Belmonte JC (2017). Ground rules of the pluripotency gene regulatory network. Nat. Rev. Genet..

[28] Takai H, Smogorzewska A, de Lange T (2003). DNA damage foci at dysfunctional telomeres. Curr. Biol..

[29] Nagaoka M (2006). E-cadherin-coated plates maintain pluripotent ES cells without colony formation. PLoS ONE.

[30] Zhao Y (2009). Telomere extension occurs at most chromosome ends and is uncoupled from fill-in in human cancer cells. Cell.

[31] Amano T (2013). Zscan4 restores the developmental potency of embryonic stem cells. Nat. Commun..

[32] Akiyama T (2015). Transient bursts of Zscan4 expression are accompanied by the rapid derepression of heterochromatin in mouse embryonic stem cells. DNA Res..

[33] Owens DM, Keyse SM (2007). Differential regulation of MAP kinase signalling by dual-specificity protein phosphatases. Oncogene.

[34] Smith AG (1988). Inhibition of pluripotential embryonic stem cell differentiation by purified polypeptides. Nature.

[35] Storm MP (2014). Zscan4 is regulated by PI3-kinase and DNA-damaging agents and directly interacts with the transcriptional repressors LSD1 and CtBP2 in mouse embryonic stem cells. PLoS ONE.

[36] Ying QL, Nichols J, Chambers I, Smith A (2003). BMP induction of Id proteins suppresses differentiation and sustains embryonic stem cell self-renewal in collaboration with STAT3. Cell.

[37] Sylva M, Moorman AF, van den Hoff MJ (2013). Follistatin-like 1 in vertebrate development. Birth Defects Res C. Embryo Today.

[38] Cuny GD (2008). Structure-activity relationship study of bone morphogenetic protein (BMP) signaling inhibitors. Bioorg. Med Chem. Lett..

[39] Dan J (2013). Roles for Tbx3 in regulation of two-cell state and telomere elongation in mouse ES cells. Sci. Rep..

[40] Eckersley-Maslin MA (2016). MERVL/Zscan4 network activation results in transient genome-wide DNA demethylation of mESCs. Cell Rep..

[41] Leitch HG (2013). Naive pluripotency is associated with global DNA hypomethylation. Nat. Struct. Mol. Biol..

[42] Ficz G (2013). FGF signaling inhibition in ESCs drives rapid genome-wide demethylation to the epigenetic ground state of pluripotency. Cell Stem Cell.

[43] Stadtfeld M, Maherali N, Breault DT, Hochedlinger K (2008). Defining molecular cornerstones during fibroblast to iPS cell reprogramming in mouse. Cell Stem Cell.

[44] Choi J (2017). Prolonged Mek1/2 suppression impairs the developmental potential of embryonic stem cells. Nature.

[45] Yagi M (2017). Derivation of ground-state female ES cells maintaining gamete-derived DNA methylation. Nature.

[46] Nakai-Futatsugi Y, Niwa H (2016). Zscan4 is activated after telomere shortening in mouse embryonic stem cells. Stem Cell Rep..

[47] Hemann MT, Strong MA, Hao LY, Greider CW (2001). The shortest telomere, not average telomere length, is critical for cell viability and chromosome stability. Cell.

[48] der-Sarkissian H, Bacchetti S, Cazes L, Londono-Vallejo JA (2004). The shortest telomeres drive karyotype evolution in transformed cells. Oncogene.

[49] Hao LY (2005). Short telomeres, even in the presence of telomerase, limit tissue renewal capacity. Cell.

[50] Pucci F, Gardano L, Harrington L (2013). Short telomeres in ESCs lead to unstable differentiation. Cell Stem Cell.

[51] Ohtsuka S, Niwa H (2015). The differential activation of intracellular signaling pathways confers the permissiveness of embryonic stem cell derivation from different mouse strains. Development.

[52] Ohtsuka S, Nishikawa-Torikai S, Niwa H (2012). E-cadherin promotes incorporation of mouse epiblast stem cells into normal development. PLoS ONE.

[53] Ben-David U (2014). Aneuploidy induces profound changes in gene expression, proliferation and tumorigenicity of human pluripotent stem cells. Nat. Commun..

[54] Baker DE (2007). Adaptation to culture of human embryonic stem cells and oncogenesis in vivo. Nat. Biotechnol..

[55] Chan YS (2013). Induction of a human pluripotent state with distinct regulatory circuitry that resembles preimplantation epiblast. Cell Stem Cell.

[56] Gafni O (2013). Derivation of novel human ground state naive pluripotent stem cells. Nature.

[57] Theunissen TW (2016). Molecular criteria for defining the naive human pluripotent state. Cell Stem Cell.

[58] Gu W (2016). Glycolytic metabolism plays a functional role in regulating human pluripotent stem cell state. Cell Stem Cell.

[59] Yang Y (2017). Derivation of pluripotent stem cells with in vivo embryonic and extraembryonic potency. Cell.

[60] Chen Z (2009). Birth of parthenote mice directly from parthenogenetic embryonic stem cells. Stem Cells.

[61] Huang J (2008). Efficient production of mice from embryonic stem cells injected into four- or eight-cell embryos by piezo micromanipulation. Stem Cells.

[62] Bao S (2009). Epigenetic reversion of post-implantation epiblast to pluripotent embryonic stem cells. Nature.

[63] Yeom YI (1996). Germline regulatory element of Oct-4 specific for the totipotent cycle of embryonal cells. Development.

[64] Tang F (2010). Tracing the derivation of embryonic stem cells from the inner cell mass by single-cell RNA-Seq analysis. Cell Stem Cell.

[65] Dan J (2014). Rif1 maintains telomere length homeostasis of ESCs by mediating heterochromatin silencing. Dev. Cell.

[66] Williams, E. S. & Bailey, S. M. Chromosome orientation fluorescence in situ hybridization (CO-FISH). *Cold Spring Harb. Protoc.* **2009**, pdb prot5269 (2009). 10.1101/pdb.prot5269 20147245

[67] Yang BX (2015). Systematic identification of factors for provirus silencing in embryonic stem cells. Cell.

[68] Weber M (2005). Chromosome-wide and promoter-specific analyses identify sites of differential DNA methylation in normal and transformed human cells. Nat. Genet..

