# Supplementary material for: Feeders facilitate telomere maintenance and chromosomal stability of embryonic stem cells
Source: Nat Commun. 2018 Jul 5;9:2620. doi: 10.1038/s41467-018-05038-2 (PMC6033898; doi:10.1038/s41467-018-05038-2)
Supplement: Supplementary file 1 — Supplementary Information [file 41467_2018_5038_MOESM1_ESM.pdf]

Supplementary Information

Feeders facilitate telomere maintenance and chromosomal stability of embryonic stem cells

Guo *et al.*

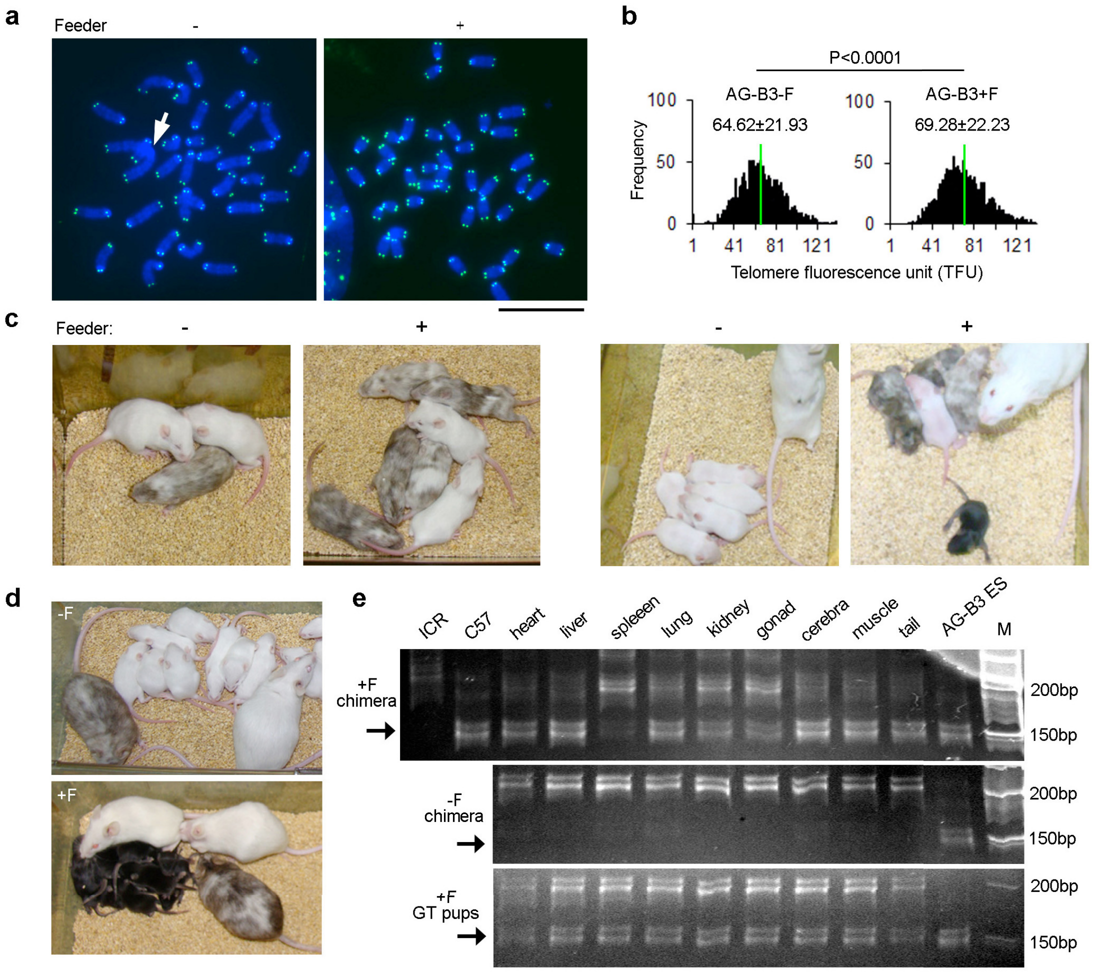

Supplementary Figure 1 Feeder supports developmental pluripotency of mouse ESCs. **a**, Telomere Q-FISH images of AG-B3 ESCs cultured on gelatin or feeder. Blue, chromosomes stained by DAPI; Green dots, telomeres. White arrow indicates chromosome fusion. Scale bar, 10 μm. **b**, Histogram shows distribution of relative telomere length displayed as TFU by Q-FISH analysis. Green line indicates medium telomere length. Mean ± s.d. of telomere length is shown above each panel. Telomere length was measured in AG-B3 ESC at passage 5 cultured with or without feeders. P value was calculated by Wilcoxon-Mann-Whitney rank sum test. **c**, Chimeric mice and all-black pups produced by injection of AG-B3 ESCs into four- or eight-cell albino ICR embryos. Representative photos from two independent experiments are shown. **d**, Chimeric mice and germline transmission (GT) pups from ESCs cultured with feeders (+F). **e**, Contributions of ESCs to various tissues of the chimera and GT pups by microsatellite genotyping analysis. DNA from ICR mice, C57 mice and AG-B3 ESC served as controls. -F, without feeders; +F, with feeders.

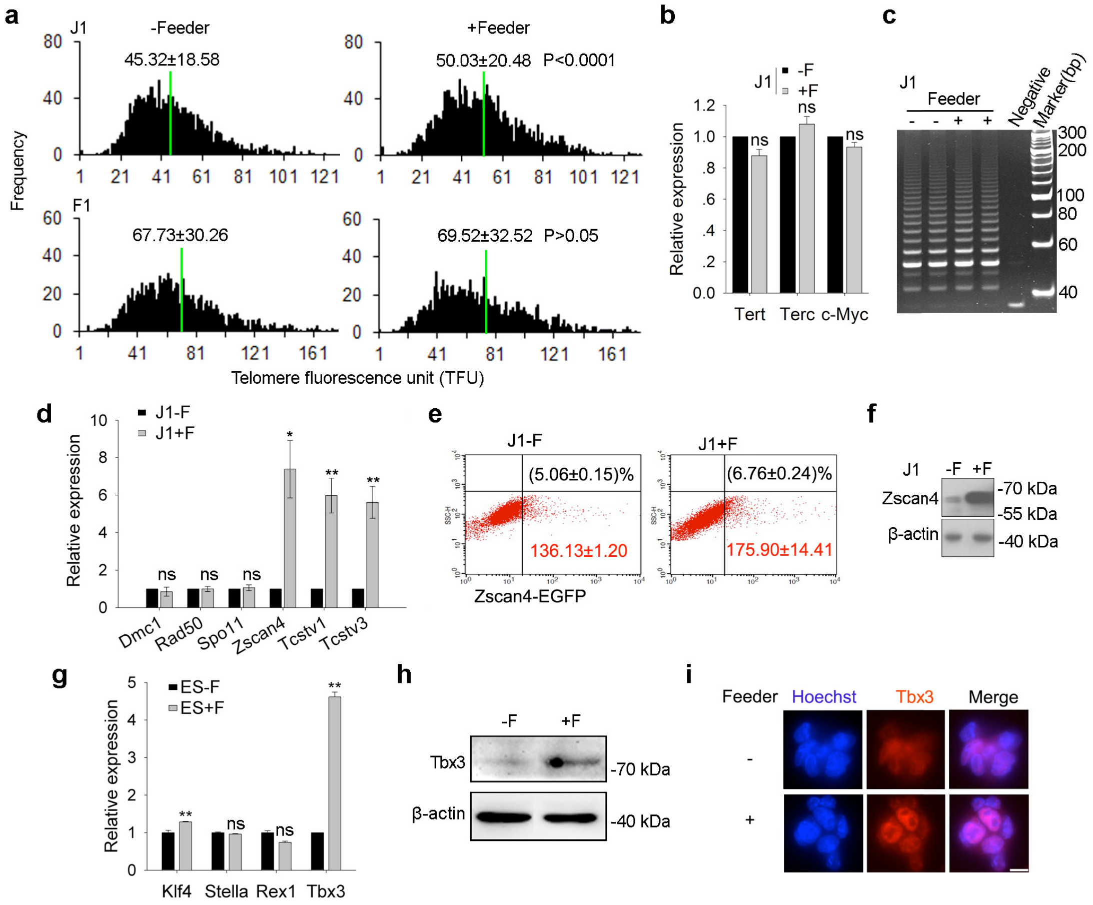

Supplementary Figure 2 Elevated expression of Zscan4 and Tbx3 in ESCs cultured with feeders. a, Histogram shows distribution of relative telomere length displayed as TFU by Q-FISH analysis of J1 and F1 ESCs. Green line indicates medium telomere length. Mean ± s.d. of telomere length is shown above each panel. Telomere length was measured in ESCs at passage 5 cultured under feeder or feeder-free conditions. Wilcoxon-Mann-Whitney rank sum test. **b**, Relative expression levels of *Tert*, *Terc* and *c-Myc* in -F and +F J1 ESCs by qPCR. **c**, Telomerase activity measured by TRAP assay. Lysis buffer served as negative control. **d**, Relative gene expression levels in -F and +F J1 ESCs. **e**, Flow cytometry quadrantal diagram indicates percentage and mean fluorescence intensity of Zscan4^{+} cells. **f**, Western blot analysis of Zscan4 protein levels. β-actin served as loading control. **g**, Expression of naive genes in -F and +F N33 ESCs. **h**, Western blot analysis of Tbx3. **i**, Immunofluorescence staining of Tbx3 in -F and +F ESCs. Scale bar, 10 μm. Mean ± s.e.m. from three independent experiments. *p<0.05, **p<0.01, ns, not significant (p>0.05). Student's t-test. -F, without feeders; +F, with feeders.

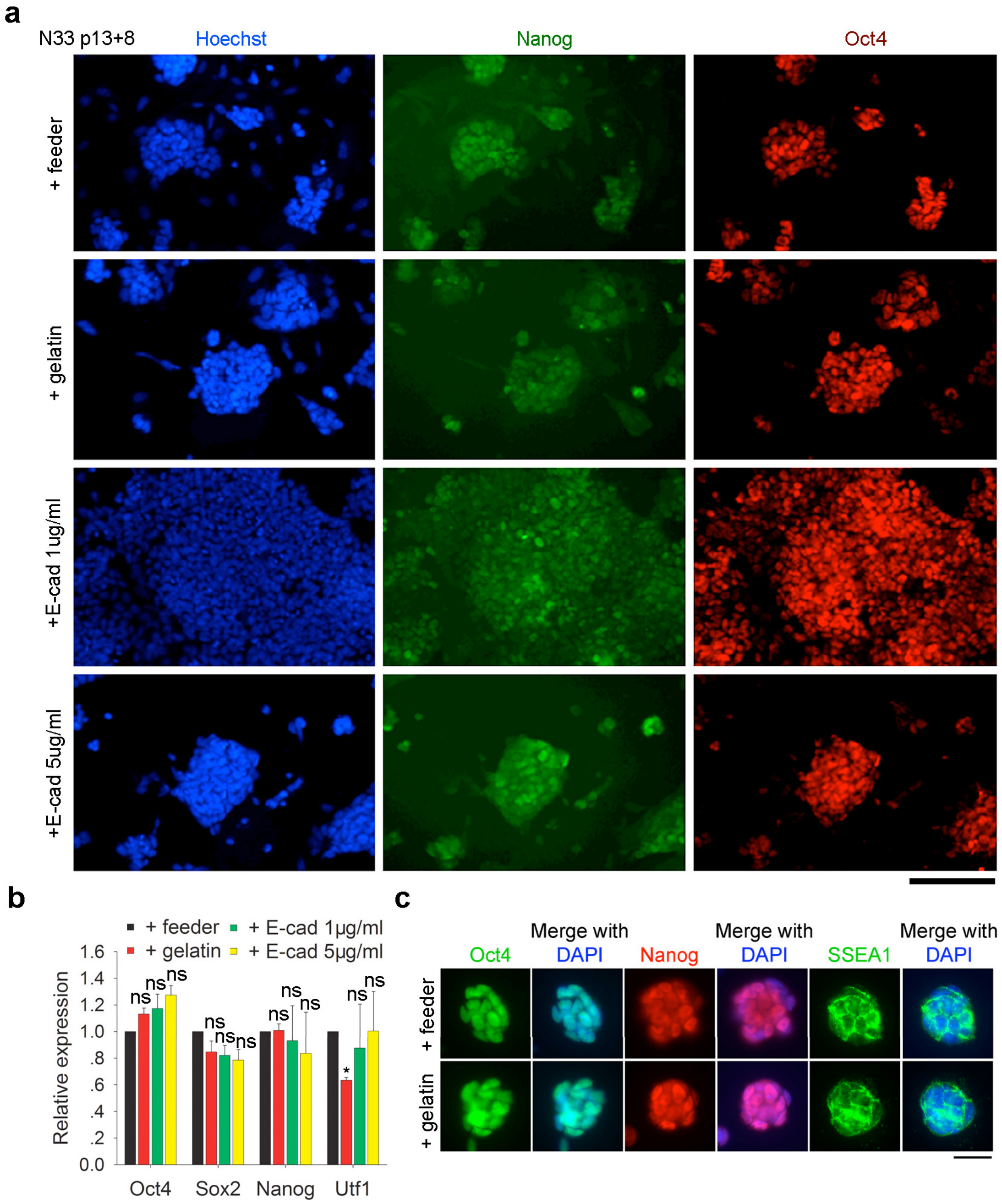

Supplementary Figure 3 Expression of pluripotency marker genes in ESCs cultured with or without feeders. a, Immunofluorescence staining of pluripotency-associated markers Nanog and Oct4. N33 ESCs were cultured on feeders or feeder-free gelatin-coated, or E-cad-fc-coated plates for 8 passages. Scale bar, 100 μm. **b**, Expression of *Oct4*, *Sox2*, *Nanog* and *Utf1* of N33 ESCs by qPCR. *$p<0.05$; ns, not significant ($p>0.05$), compared with ESCs cultured on feeders. ANOVA with Fisher's protected least-significant difference (PLSD) analysis. **c**, Immunofluorescence of Oct4, Nanog and SSEA1 under higher magnification. Scale bar, 20 μm.

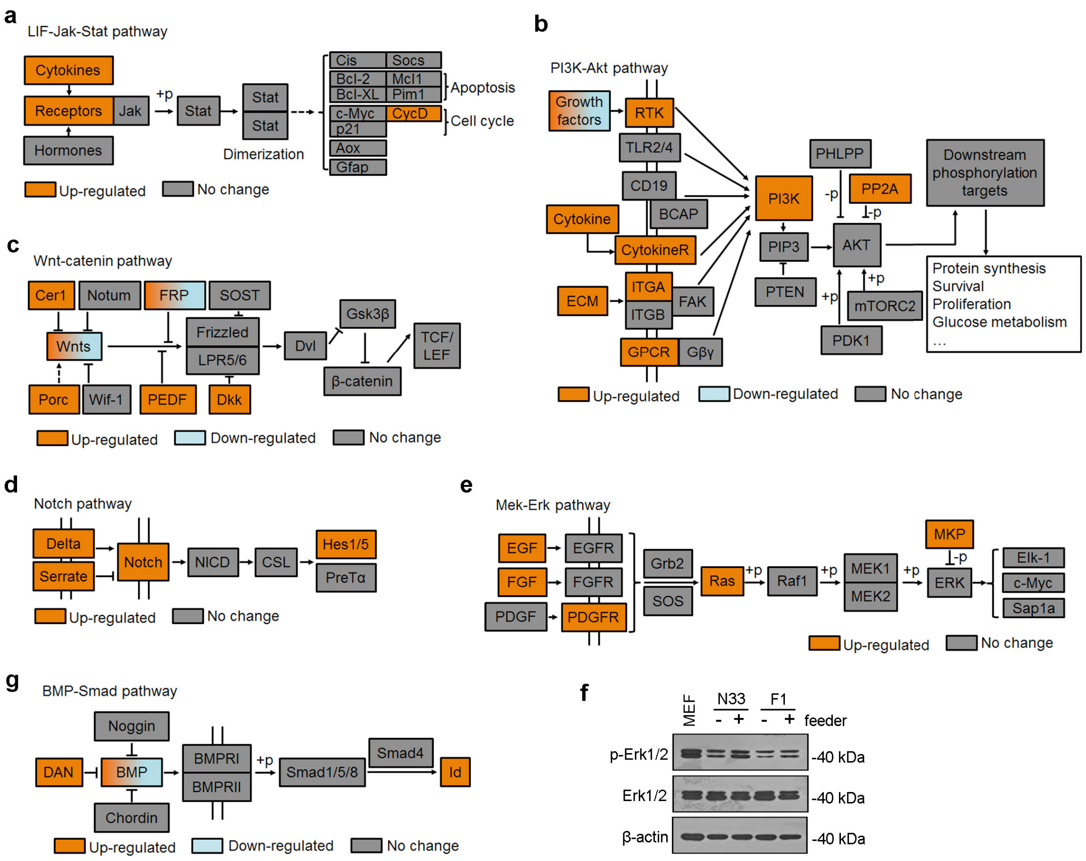

Supplementary Figure 4 Signal pathways by RNA-seq data analysis. a, Cytokines (e.g. ILs and IFNs) and receptors (e.g. ILRs) involved in LIF pathway up-regulated in feeder+ESCs compared with feeder-free ESCs. Most of LIF targeting genes are not changed in their expression levels except for CycD. **b**, Cytokines, ECMs, some growth factors and their corresponding receptors upstream PI3K-Akt are activated in ESCs cultured with feeders. However, serine/threonine-protein phosphatase PP2A is also up-regulated, which may partly explain the decreased phosphorylation levels of Akt at Thr308 and Ser473 in feeder+ESCs. **c**, Both activating factors (e.g. Wnts) and inactivating ones (e.g. PEDF and Dkk) are up-regulated in feeder+ESCs compared with feeder-free ESCs, and expression of β-catenin not altered. **d**, Notch signal pathways. Notch targeting genes, Hes1 and Hes5 are up-regulated in feeder+ESCs. **e**, In Mek-ERK pathway, growth factors (e.g. EGFs and FGFs) and Ras are activated, however, MKPs (including DUSP1, DUSP9, DUSP10) also up-regulated in ESCs cultured with feeders. **f**, Levels of p-Erk1/2 and Erk1/2 protein in -F and +F ESCs. Two repeats. β-actin served as loading control. **g**, BMP-Smad pathway. BMP-Smad targeting Id genes mostly are activated in ESCs cultured with feeders.

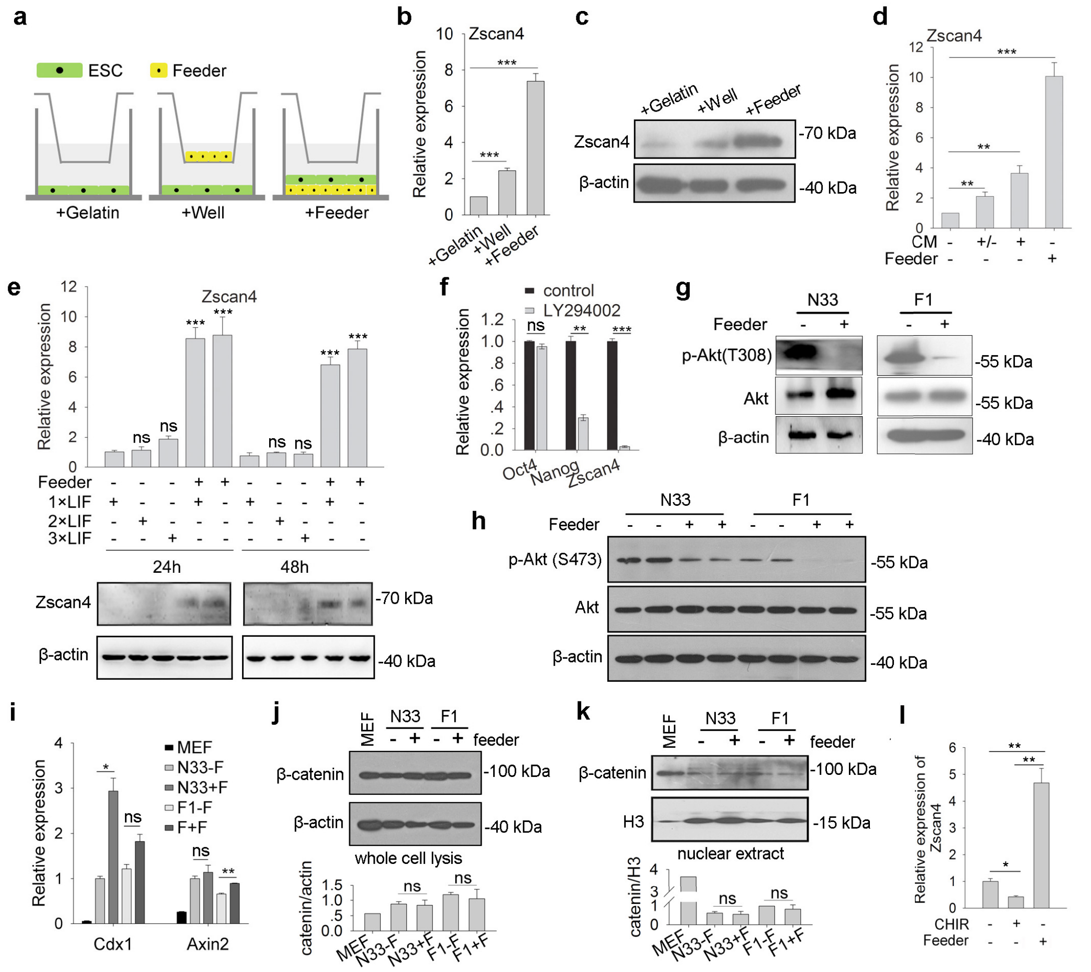

Supplementary Figure 5 Signal transduction in ESCs cultured without (-F) or with feeders (+F). **a**, Schematic diagram of trans-well assay. **b**, Expression of *Zscan4* by qPCR. **c**, Western blot analysis of Zscan4 protein levels. **d**, Expression level of *Zscan4* is increased in feeder-conditioned medium (CM). +/-, CM was diluted with same volume of ESC medium. **e**, mRNA and protein levels (bottom panel) of Zscan4 in ESCs treated with LIF from 1000 U/ml (1×), 2000 U/ml (2×) to 3000 U/ml (3×) for 24 h and 48 h. **f**, LY294002 (10 μM) inhibits expression of *Nanog* and *Zscan4*, but not *Oct4*. **g,h**, Decreased phosphorylation levels of Akt at Thr308 (g) and Ser473 (h) in +F ESCs. **i**, Expression of Wnt targeting genes, *Cdx1* and *Axin2*. **j,k**, Protein levels of total (j) and nuclear (k) β-catenin in -F and +F ESCs by Western blot analysis. Bottom panel, Quantity using ImageJ software of β-catenin protein levels relative to β-actin or H3 served as internal control. **l**, Relative expression level of *Zscan4* by qPCR analysis following treatment of N33 ESCs with CHIR99021 (CHIR, 3 μM) for three passages. Data represent mean ± s.e.m. from three independent experiments. *p<0.05, **p<0.01, ***p < 0.001, ns, not significant (p>0.05). ANOVA with PLSD for b,d,e,l, and Student's t-test for i-k.

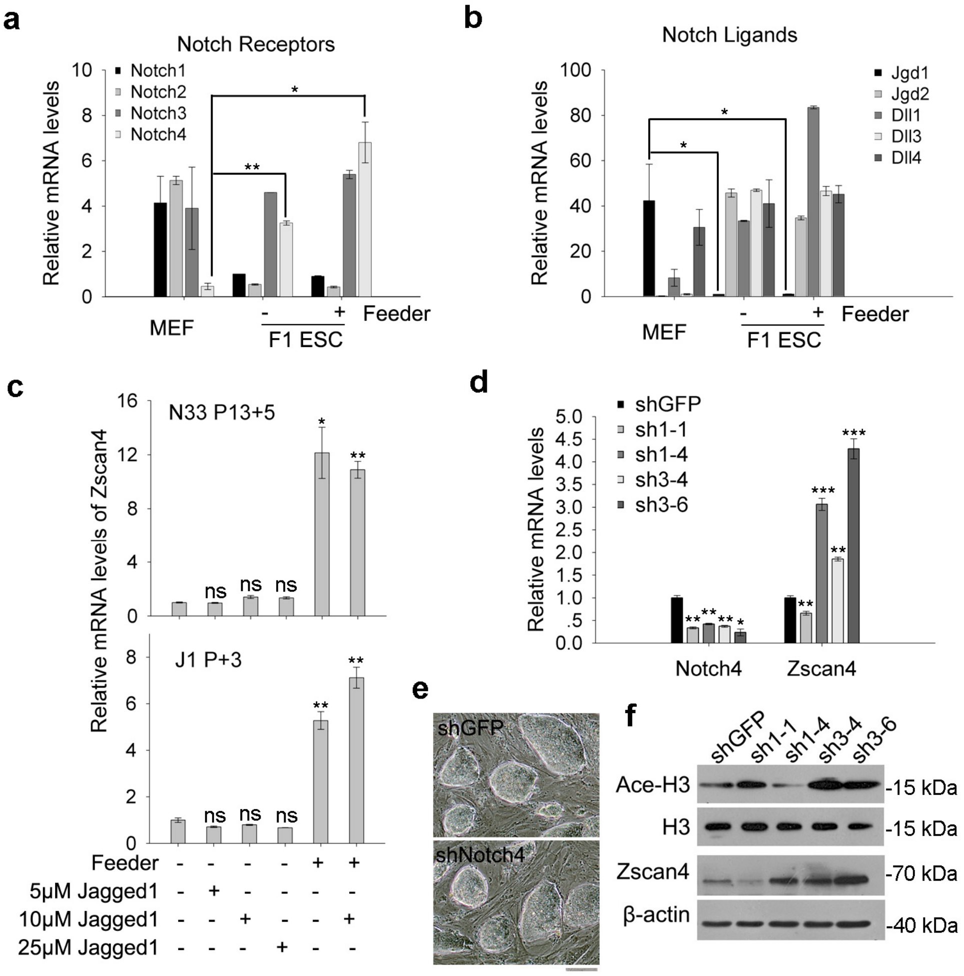

Supplementary Figure 6 Notch signaling and up-regulation of *Zscan4* following knockdown of Notch4 by shRNAs. a-b, Relative expression level by qPCR analysis of Notch receptors (a) and ligands (b) in MEFs, and ESCs cultured with or without feeders. **c**, Expression of *Zscan4* in ESCs after treatment with Jagged1 peptides. **d**, Expression by qPCR analysis of *Zscan4* and *Notch4* after *Notch4* knockdown. **e**, Representative images of ESCs after transfection with a control shRNA construct (shGFP) or shRNAs targeting *Notch4*. Scale bar, 100 μm. **f**, Protein levels of Zscan4 and Histone H3 acetylation by Western blot. Data represent Mean ± s.e.m. from three independent experiments. *p<0.05, **p<0.01, ***p < 0.001, ns, not significant (p>0.05), compared between two selected groups for a and b, or compared with control groups for c and d. ANOVA with PLSD was used for the statistical analysis.

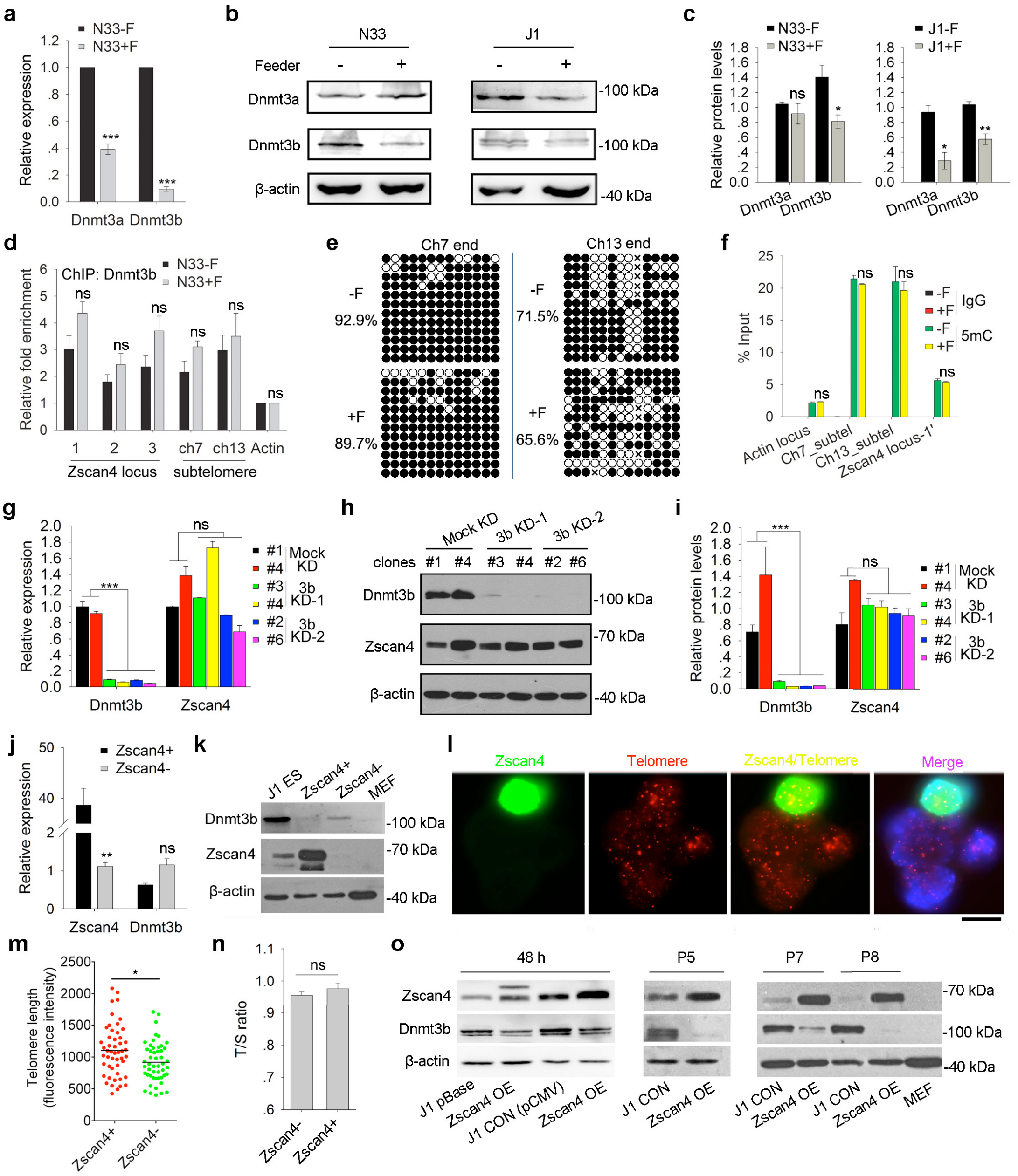

Supplementary Figure 7 Feeders decrease *Dnmt3b* expression in ESCs. a, Expression of *Dnmt3a* and *Dnmt3b* by qPCR. **b**, Western blot analysis of Dnmt3a and Dnmt3b protein levels in N33 and J1 ESCs. **c**, Relative protein levels of Dnmt3a and Dnmt3b normalized to β-actin by ImageJ software. **d**, ChIP-qPCR analysis of Dnmt3b occupancy at three proximal regions of *Zscan4c* promoter, subtelomere of chr7 and chr13. The β-actin locus served as control. **e**, Bisulfite sequencing analysis of DNA methylation at subtelomeric regions of chr7 and chr13. White circles indicate unmethylated cytosines; Black circles methylated cytosine and crosses mutation of cytosine. **f**, MeDIP-qPCR analysis of 5mC levels at subtelomeres of chr7, chr13 and *Zscan4c* promoter in -F and +F ESCs. The β-actin locus served as control. **g**, Expression of *Dnmt3b* and *Zscan4* after stable knockdown (KD) of *Dnmt3b* for 9 passages. Two different shRNAs sequences against *Dnmt3b* were used. #, clones

picked. **h**, Western blot analysis of Dnmt3b and Zscan4 protein levels. **i**, Relative protein levels of Dnmt3b and Zscan4 in mock KD and *Dnmt3b* KD ESCs normalized to β-actin by ImageJ software. **j,k**, Expression levels of Zscan4 and Dnmt3b in $Zscan4^+$ and $Zscan4^-$ ESCs sorted by flow cytometry followed by qPCR (j) and by Western blot analysis (k). J1 ESCs and MEF served as positive and negative controls for *Zscan4* expression, respectively. **l**, Representative IF-FISH images of Zscan4 (green) and telomere (red). Scale bar, 10 µm. **m**, Relative telomere lengths (fluorescence intensity) of Zscan4+ and Zscan4- ESCs estimated by ImageJ. 50 cells were counted for each group. **n**, Relative telomere length shown as T/S ratio by qPCR. **o**, Changes of Dnmt3b protein levels following overexpression of *Zscan4*. Overexpression of *Zscan4* using two different vectors (Pbase or Pcmv) for 48 h immediately reduces Dnmt3b protein levels and stable overexpression of *Zscan4* also leads to reduced levels of Dnmt3b after several passages. Mean ± s.e.m. from three independent experiments. $*p < 0.05$, $**p<0.01$, $***p < 0.001$, ns, not significant ($p>0.05$). Student's t-test.

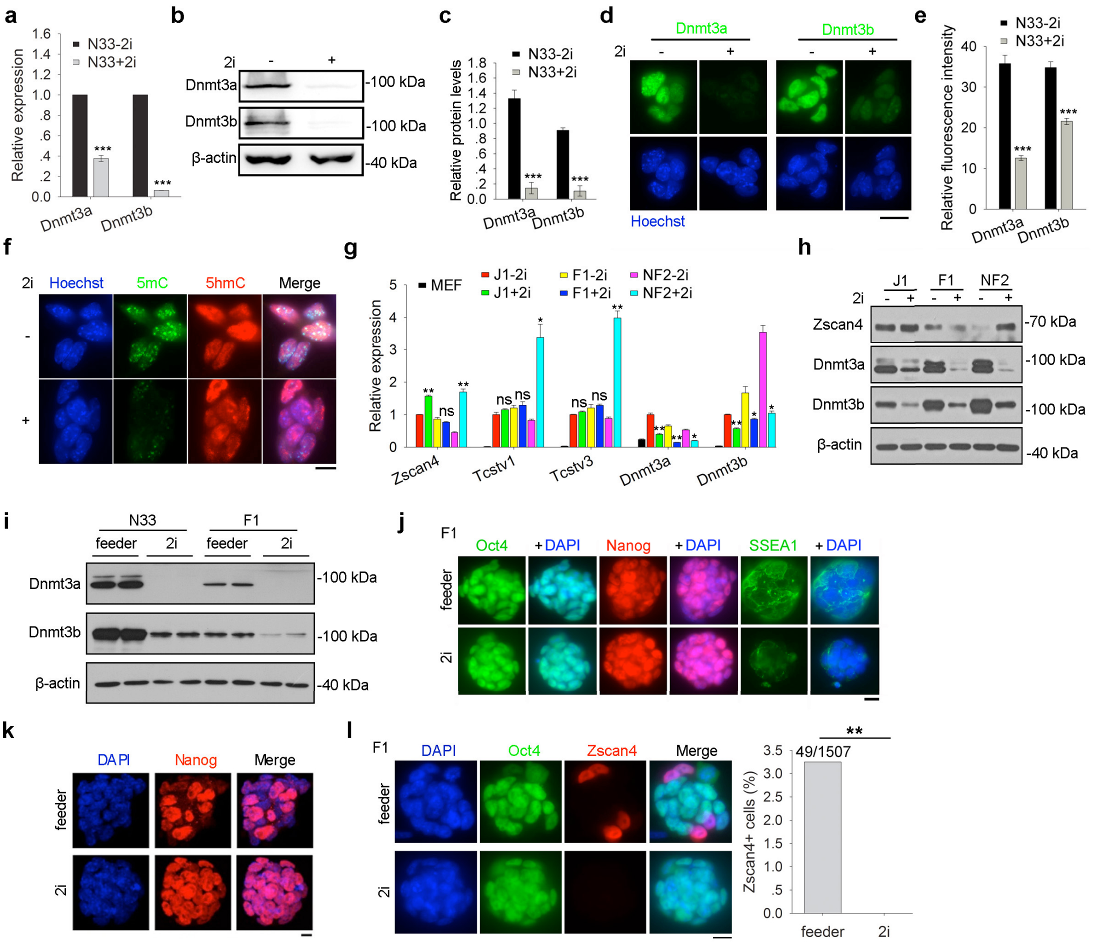

Supplementary Figure 8 2i repress Dnmt3a/3b, SSEA1 and Zscan4. 2i, PD0325901 (Mek inhibitor) and CHIR99021 (Gsk3β inhibitor). **a-h**, ESCs cultured under serum+feeder conditions added with 2i (+2i) or DMSO control (-2i) for 5 passages. **a**, Expression by qPCR analysis. **b**, Western blot analysis. **c**, Relative protein levels of Dnmt3a/b normalized to β-actin by ImageJ software. **d**, Immunofluorescence of Dnmt3a and Dnmt3b. Scale bar, 10 μm. **e**, Relative fluorescence intensity of Dnmt3a and Dnmt3b by ImageJ. **f**, Immunofluorescence of DNA methylation by 5mC and DNA hydroxymethylation by 5hmC. Scale bar, 5 μm. **g**, Gene expression by qPCR of three ESC lines. P values, compared with ESCs cultured without 2i (-2i). **h**, Western blot of Dnmt3a, Dnmt3b and Zscan4. **i**, N33 and F1 ESCs cultured for 5 and 3 passages, respectively, in serum+feeder+LIF or N2B27+2i+LIF conditions and protein levels of Dnmt3a/3b by Western blot. **j**, Immunofluorescence of Oct4, Nanog and SSEA1 in F1 ESCs (3 passages). Scale bar, 10 μm. **k**, Immunofluorescence of Nanog by Confocal Laser Scanning Microscope indicating more homogenous expression of Nanog in 2i culture. Scale bar, 10 μm. **l**, Immunofluorescence of co-staining of Oct4 and Zscan4 in F1 ESCs. Scale bar, 10 μm. Right panel, Percentage of $Zscan4^{+}$ cells in approximately 1,500 ESCs counted. Mean ± s.e.m. from three independent experiments. *$p < 0.05$, **$p<0.01$, ***$p < 0.001$, ns, not significant ($p>0.05$). $\chi 2$ test for l, and Student's t-test for a, c, e, g.

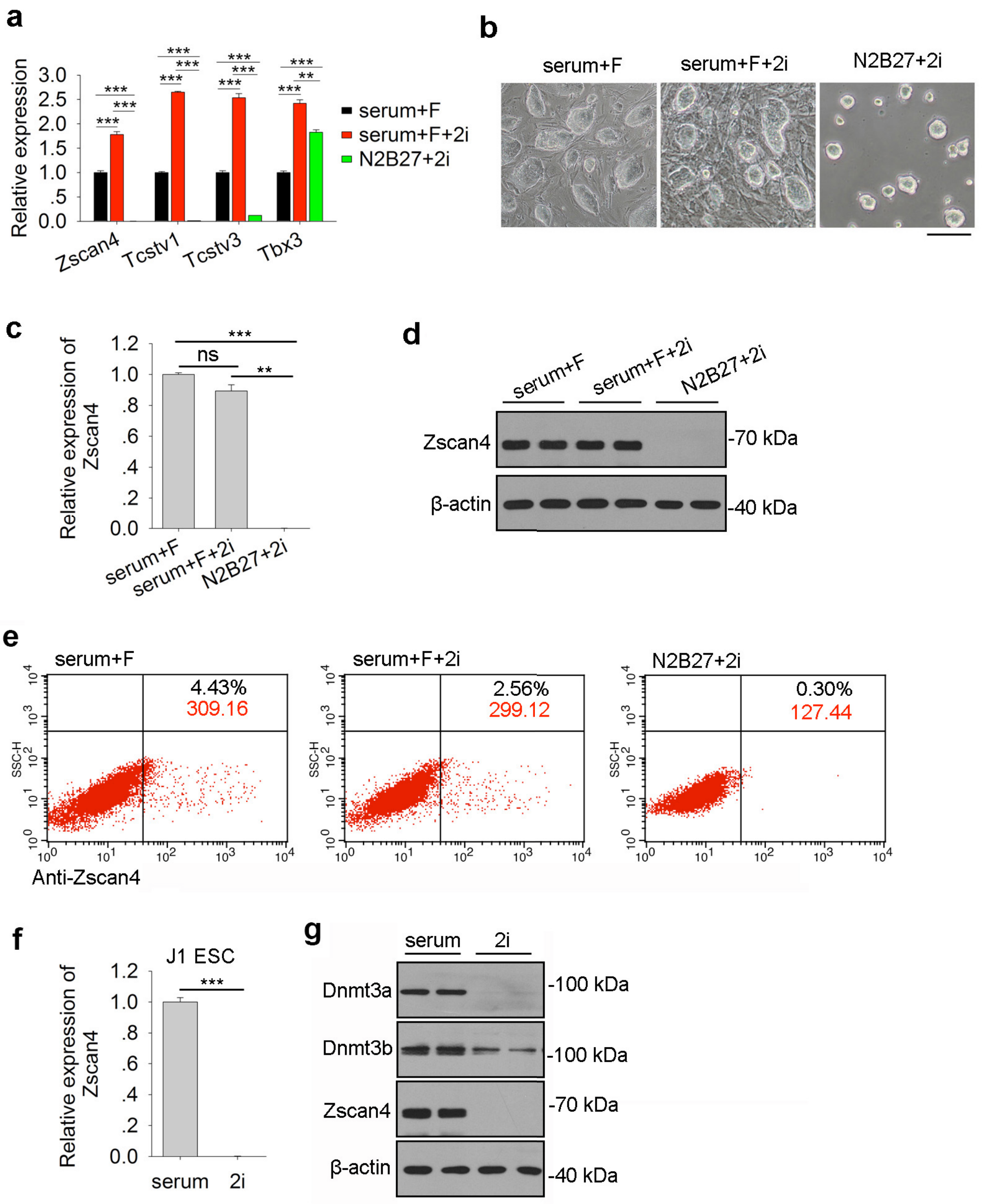

Supplementary Figure 9 ***Zscan4* is repressed in ESCs cultured in N2B27+2i/L medium. a**, Expression by qPCR of indicated genes of N33 ESCs cultured for 5 passages in three conditions. **b**, Morphology of N33 ESC colonies under bright field with phase-contrast optics. Scale bar, 100 μm. **c**, Relative expression of *Zscan4* as measured by qPCR analysis of ESCs cultured for four passages. **d**, Zscan4 protein levels by Western blot. **e**, Flow cytometry quadrantal diagram indicating proportion and mean fluorescence intensity of $Zscan4^{+}$ cells in a ESC population. **f**, qPCR analysis of *Zscan4* mRNA levels of J1 ESCs cultured in serum+feeder and 2i/L conditions for four passages. **g**, Western blot analysis of Dnmt3a, Dnmt3b and Zscan4 protein levels. β-actin served as loading control. Mean ± s.e.m. from three independent experiments. $^{**}p < 0.01$, $^{***}p < 0.001$, ns, not significant ($p > 0.05$). ANOVA with PLSD for a,c, and Student's t-test for f.

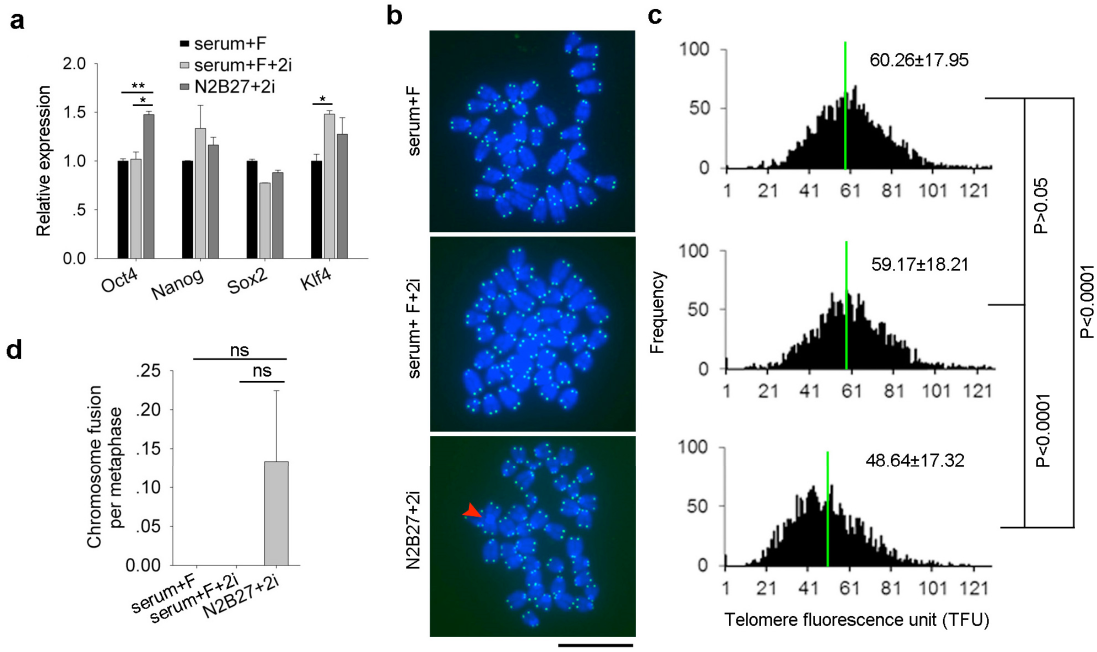

Supplementary Figure 10 Shorter telomeres in N33 ESCs cultured in N2B27+2i/L media compared with cultures with feeders. **a**, Expression levels of pluripotency-associated genes by qPCR of ESCs cultured for five passages. Mean ± s.e.m. from three independent experiments. *p < 0.05, **p < 0.01. ANOVA with PLSD. **b**, Representative telomere Q-FISH images. Blue, chromosomes stained by DAPI; Green dots, telomeres. Red arrowhead indicates chromosome fusion. **c**, Histogram displaying distribution of relative telomere length shown as TFU. Green line indicates medium telomere length. Mean ± s.d. of telomere length is shown at right corner of each panel. Wilcoxon-Mann-Whitney rank sum test. **d**, Frequency of chromosomal fusion per metaphase. ns, not significant (p>0.05). ANOVA with PLSD.

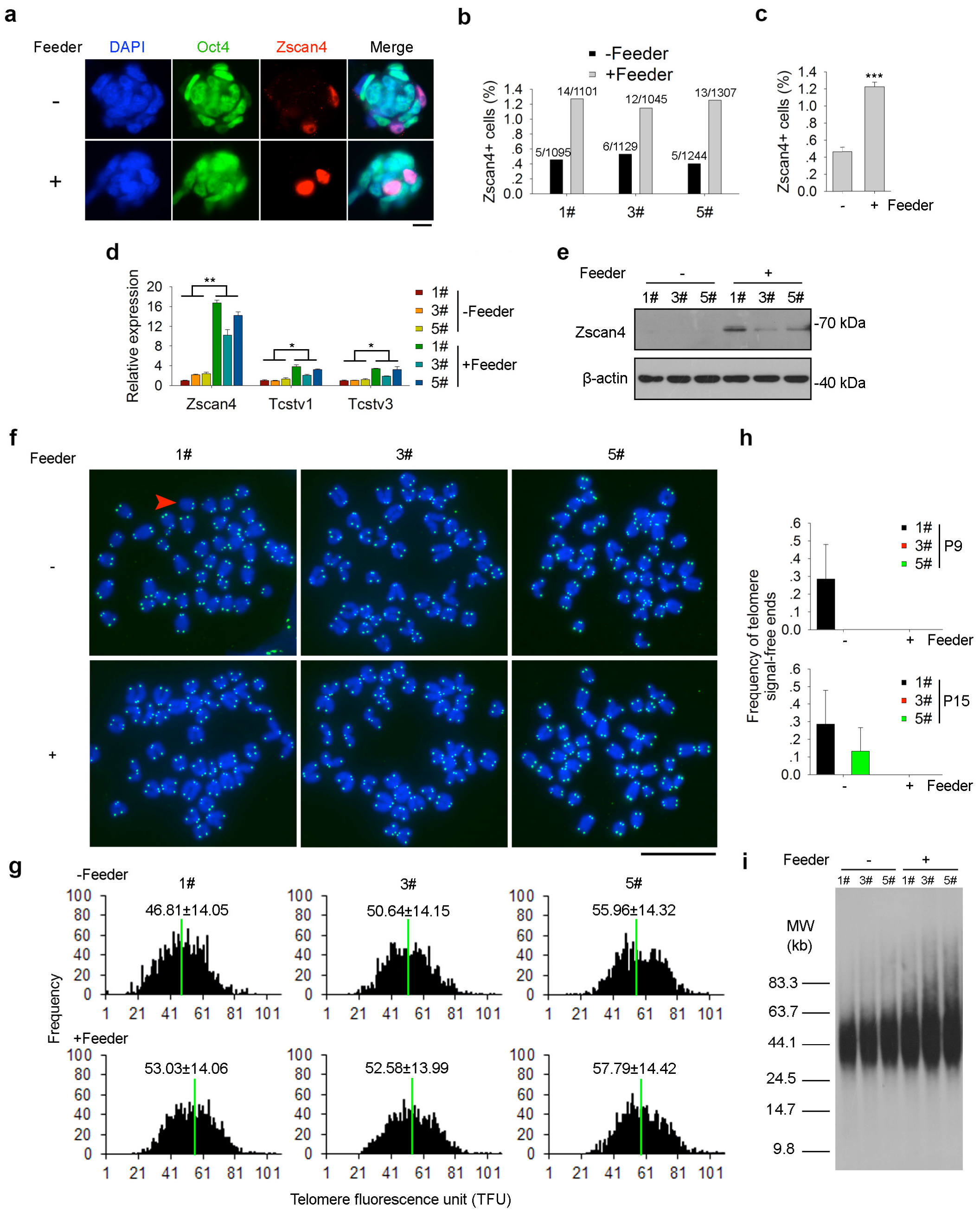

Supplementary Figure 11 Zscan4 expression and telomere elongation in 129xC57/B6 ESCs cultured with feeders compared to those without feeders. ESCs were initially maintained in serum+feeder+LIF condition for 4 passages, and then were split onto plates with or without feeders for 5 passages (P9) or 11 passages (P15). Three independent ESC lines (1#, 3#, 5#) were used for analysis of gene expression and telomere lengths. 129xC57/B6 ESCs exhibited high developmental pluripotency as determined by TEC assay. At passage 5, 23 embryos were transferred following injection of the ESCs into tetraploid albino embryos, and of 8 live pups born, four ESC-pups live to adults (over 5 weeks). At passage 14, 7 live pups were born from 40 embryos and 6 of them were still alive. **a**, Immunofluorescence co-staining of Oct4 and Zscan4. Scale bar, 10 μm. **b**, Percentage of Zscan4^{+} cells for

each cell line (shown are number of ESCs counted). **c**, Percentage of Zscan4$^+$ cells for all cell lines. Mean ± s.e.m, ***p<0.001. Student's t-test. **d**, Relative expression levels by qPCR of *Zscan4*, *Tcstv1* and *Tcstv3*. Mean ± s.d, *p<0.05, **p<0.01. Student's t-test. **e**, Western blot of Zscan4 in ESCs cultures with or without feeders. **f**, Representative telomere Q-FISH images of ESCs cultured on gelatin or feeders at P9. Blue, chromosomes stained by DAPI; Green dots, telomeres; Red arrowhead indicate telomere signal-free ends. **g**, Histogram shows distribution of relative telomere length displayed as TFU by Q-FISH analysis. Green line indicates medium telomere length. Mean ± s.d. of telomere length is shown above each panel. Scale bar, 10 μm. **h**, Frequency of telomere signal-free ends indicative of shortest telomere per metaphase at P9 and P15. Mean ± s.e.m. **i**, Telomere length distribution shown as TRF by Southern blot analysis at P9.

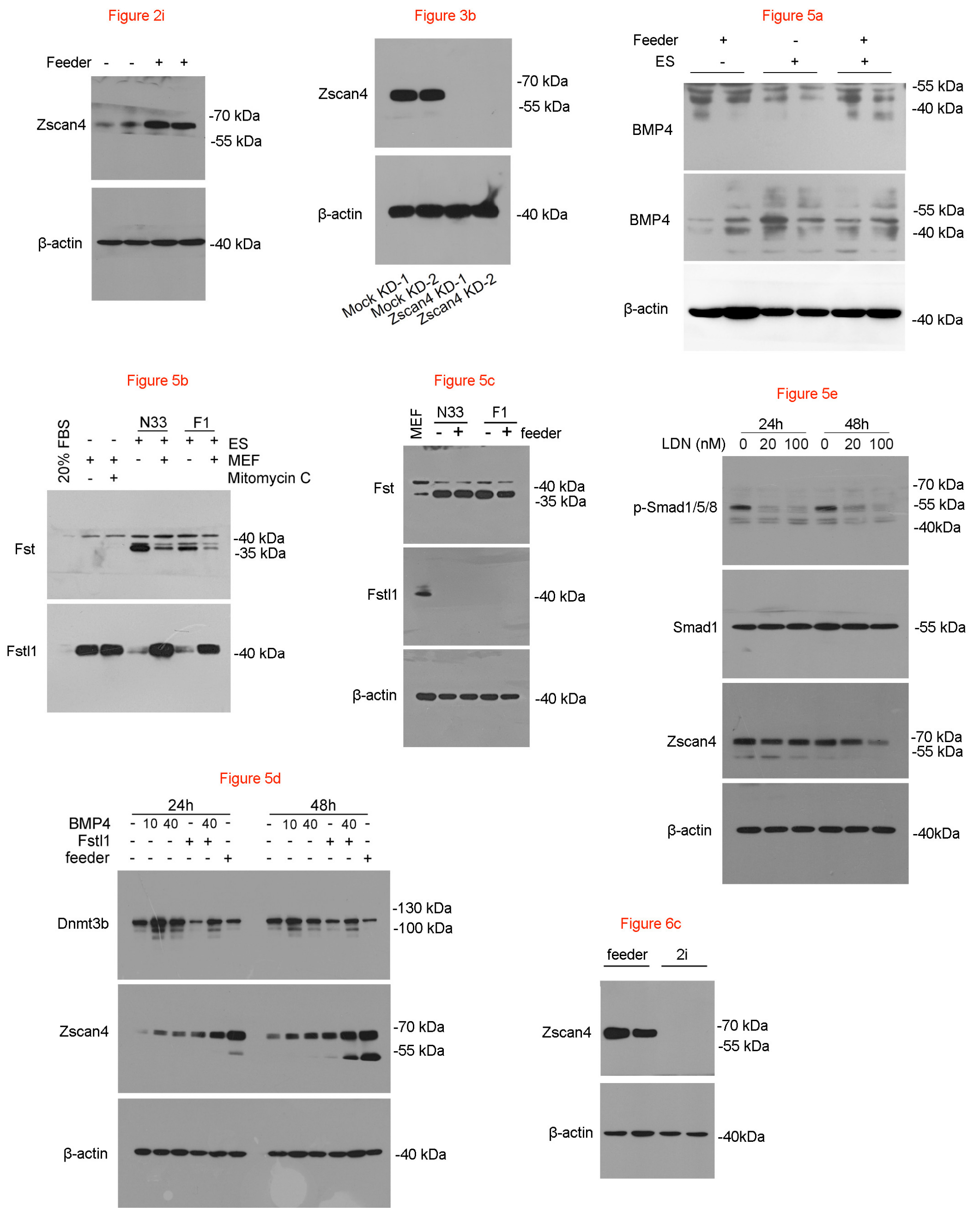

Supplementary Figure 12 Uncropped scans of Western blot with molecular weight markers. Related figure is indicated in red.

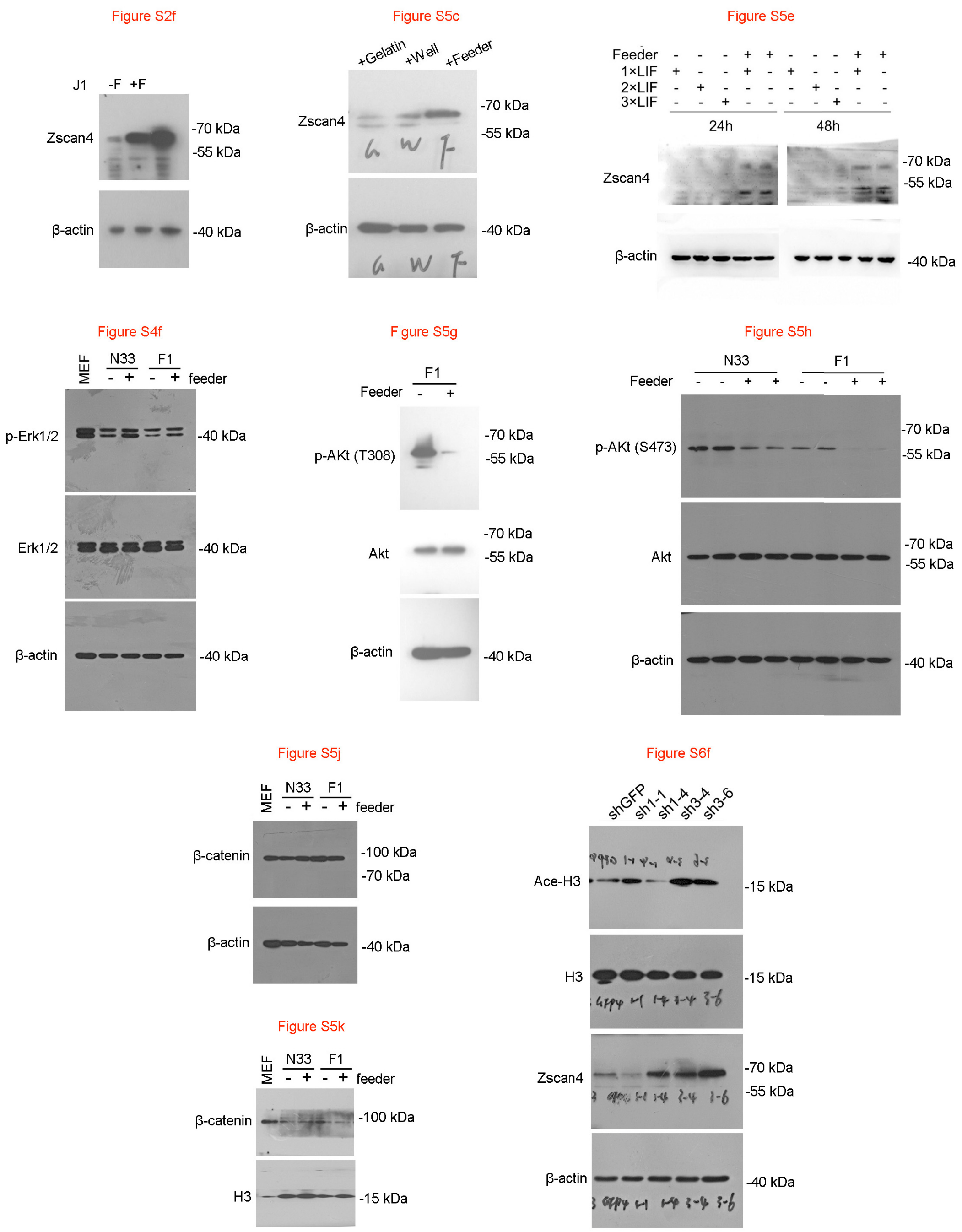

Supplementary Figure 12 (continued) Uncropped scans of Western blot with molecular weight markers. Related figure is indicated in red.

Figure S7b

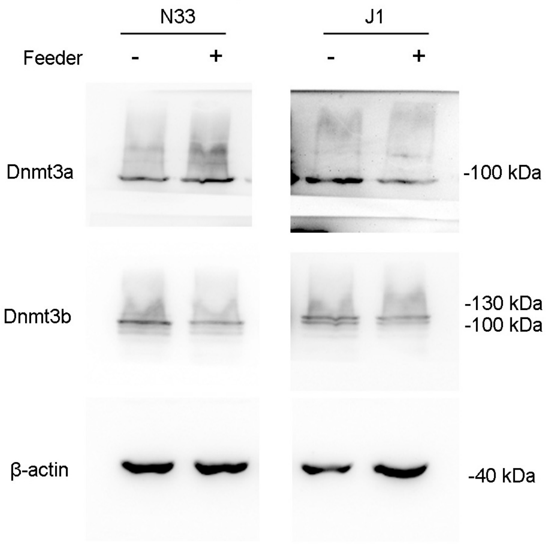

Figure S7h

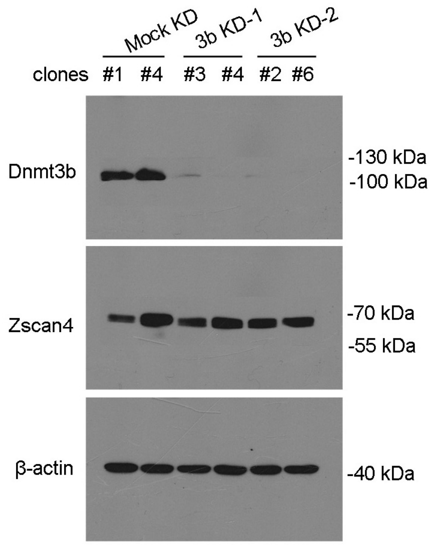

Figure S8b

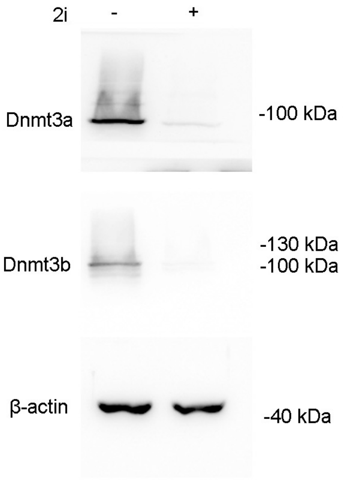

Figure S8h

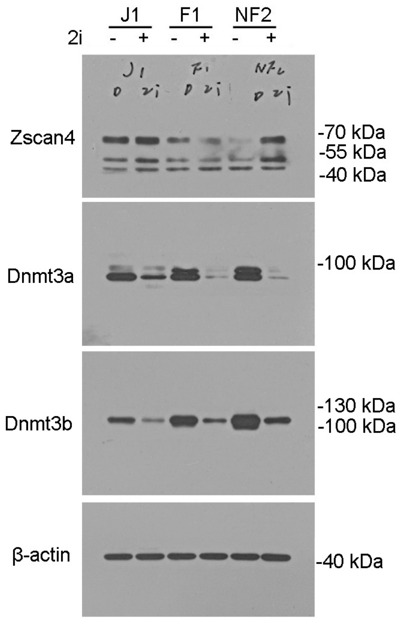

Figure S8i

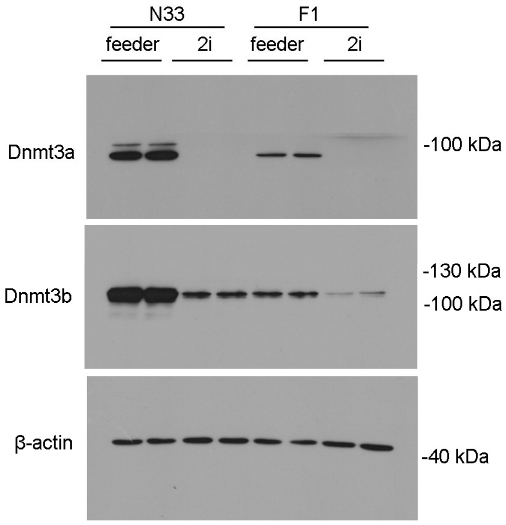

Supplementary Figure 12 (continued) Uncropped scans of Western blot with molecular weight markers. Related figure is indicated in red.

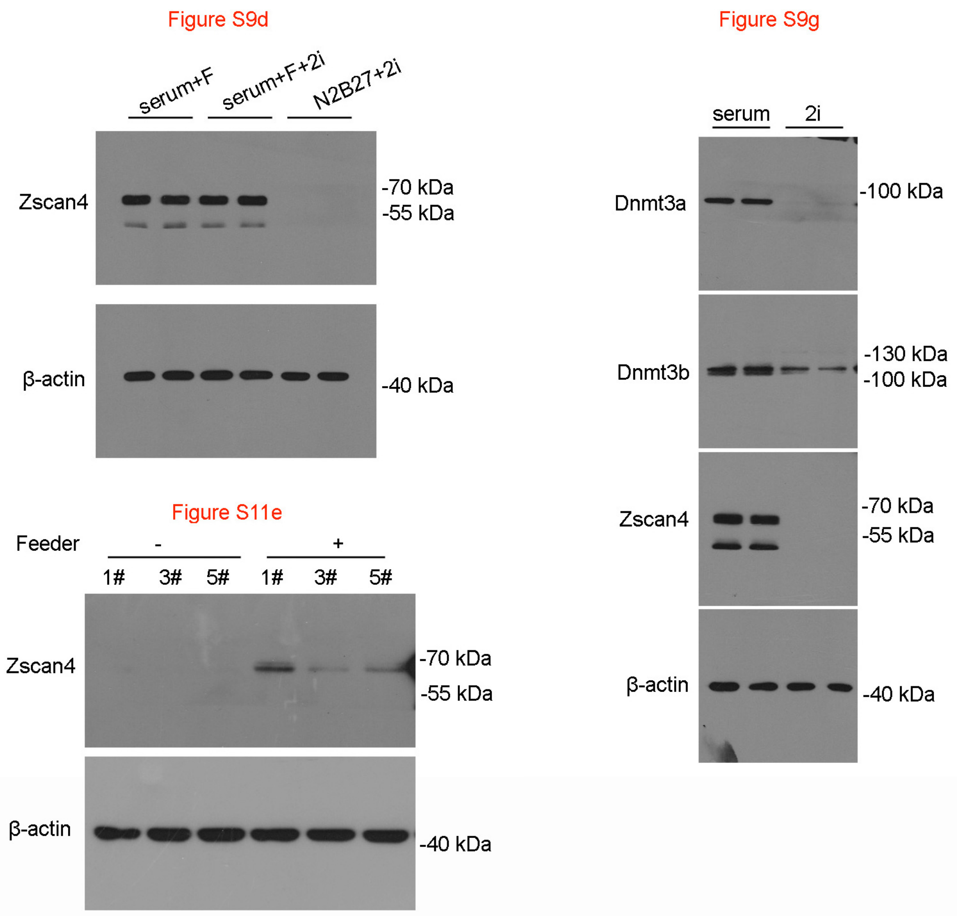

Supplementary Figure 12 (continued) Uncropped scans of Western blot with molecular weight markers. Related figure is indicated in red.

Supplementary Table 1 Efficiency in production of chimeras

Cultures	No. embryos Injected	Pups born (%)	No. Chimeras (%)	No. GT mice (%)
-F	39	8(20.5)	1(12.5)	0
+F	61	12(19.7)	9(75)	2(16.7)

ESC were injected into four- or eight-cell embryos. AG-B3 ESCs were cultured with or without feeder for 4 passages prior to injection. -F, without feeders; +F, with feeders; GT: germline transmission.

Supplementary Table 2 Primers for qPCR analysis

Genes	Forward	Reverse
Oct4	TTGGGCTAGAGAAGGATGTGGTT	GGAAAAGGGACTGAGTAGAGTGTGG
Sox2	GCACATGAACGGCTGGAGCAACG	TGCTGCGAGTAGGACATGCTGTAGG
Nanog	TTGCTTACAAGGGTCTGCTACT	ACTGGTAGAAGAATCAGGGCT
Utf1	TTCGCCGCCGCTCTACT	CAGGGGCAGGTTCGTCATT
Tert	ACTGGTGGAGATCATCTTTCTGGG	ACCTGAGGAGTCTGACATATTGGC
Terc	CATTAGCTGTGGGTTCTGGTCT	TCCTGCGCTGACGTTTGTTT
Dmc1	CAGATCCAGGAGCAACTATGA	CGATCCTCAGTTCTCCTCTT
Rad50	TGATAAGTTGTCTTGGGGTTTCC	CTGTGTCTGACGCACCTGT
Spo11	GCTGGACAGCATCCTGAAGAGG	GGGTAAGTACACTCTGGACA
Zscan4	AAATGCCTTATGTCTGTTCCCTATG	TGTGGTAATTCCTCAGGTGACGAT
Tcstv1	TGAACCCTGATGCCTGCTAAGACT	AGATGGCTGCAAAGACACAACTGC
Tcstv3	AGAAAGGGCTGGAACTTGTGACCT	AAAGCTCTTTGAAGCCATGCCCAG
Klf4	CCATCGGACCTACTTATCTGC	AAAACCTCAAACCAAAACCC
Stella	CCCAATGAAGGACCCTGAAAC	AATGGCTCACTGTCCCGTTCA
Rex1	ACGAGTGGCAGTTTCTTCTTGGGA	TATGACTCACTTCCAGGGGGCACT
Tbx3	CCACCCGTTCCTCAATTTGAACAG	CGGAAGCCATTGATGGTAAAGCTG
Dnmt3a	CAGCACCATTCCTGGTCATGCAAA	TCCTGTGTGGTAGGCACCTGAAA
Dnmt3b	GCACAACCAATGACTCTGCTGCTT	AGGACAAACAGCGGTCTTCCAGAT
Cdx1	CAACGCCTAGAGCTGGAAAA	GATCTTTACCTGCCGCTCTG
Axin2	CCAACACTTTGGCACAGCTA	TTCCTGTCCCTCTGCTGACT
Notch1	AGTGTCAGAGGCCAGCAAGAAGAA	TGATTGTCGTCCATCAGAGCACCA
Notch2	TTCATGACATGCAGCCTTTGGCTC	AGGAACTGGATGTAACCTGCCCAA
Notch3	AAATACAGGGCAGAAGGTGGGAGT	AAAGGACTGTGACCAGGTGCCATA
Notch4	CCAGAATGCGAGACAGAACTG	CAGGTGCAGGAATAGCCCTC
Jagged1	TAGTGAATGTGCCCTGGTGTCCAT	ATGATCCTAAGGCTGCCATCACCA
Jagged2	CGTATGGTGGACACGAAAGCG	GGTTGCGGATGGGATTGAGC
Delta-like1	ACGGAGAAGGTTGCTCTGTGTTCT	TCATCACACCCTGGCAGACAGATT
Delta-like3	AGTTCGTCGGCTGACTGGAATCAT	AGGATCAGGCCTCTCGTGCATAAA
Delta-like4	TTCGCCAAATCTTACCCACA	CACCAACTCCTTCGTCGTCA
Gapdh	TCAACAGCAACTCCCACTCTTCCA	ACCACCCTGTTGCTGTAGCCGTAT

Supplementary Table 3 Antibodies

Antibody	Source	Cat. No.	Dilution (IF)	Dilution (WB)
Nanog	Abcam	ab80892	1/200	
Oct3/4	Santa Cruz Biotechnology	sc-5279	1/200	
SSEA1	Millipore	MAB4301	1/200	
Zscan4	Millipore	AB4340	1/200	1/1000
Trf1	Alpha Dragrostic	TRF12-S	1/10000	
γH2AX	Millipore	05-636	1/200	
β-actin	Abmart	P30002		1/1000
Tbx3	Santa Cruz Biotechnology	sc-31657	1/200	1/500
p-Akt (Thr308)	Cell Signalling	2965		1/1000
p-Akt (Ser473)	Cell Signalling	4060		1/1000
pan-Akt	Cell Signalling	4691		1/1000
β-catenin	Abcam	ab16051		1/2000
p-ERK1/2	Cell Signalling	9101		1/1000
ERK1 (K23)	Santa Cruz Biotechnology	sc-94		1/2000
BMP4	Abclonal	A1565		1/500
Follistatin	Abcam	ab64490		1/1000
Follistatin like 1	R&D Systems	AF1738		1/1000
p-Smad1/5/8	Cell Signalling	9511		1/1000
Smad1	Cell Signalling	9743		1/1000
Ace-H3	Millipore	06-599		1/3000
H3	Abcam	ab1791		1/5000
Dnmt3a	Abcam	ab13888		1/500
Dnmt3b	Abcam	ab13604		1/1000
5mC	Millipore	NA81 (for IF)	1/100	
5mC	Active Motif	39649 (for MeDIP)		
5hmC	Active Motif	39769		

IF: immunofluorescence; WB: Western blot; MeDIP: methylated DNA immunoprecipitation

Supplementary Table 4 ChIP-qPCR primers for Zscan4 loci and sub-telomeres

Primer	Forward	Reverse
Zscan4 locus-1	CAGTGAGGTGGAGGAATAGG	AAGCTTCCAAAAGGCAATAT
Zscan4 locus-1’	GGGCTGAGCTGAGACTGATGACC	CACTTCAGCATCCACACTGGAGAGA
Zscan4 locus-2	GCATTATCTGTTCCTCTGGGTC	AACTCCTGTTCCTGGGTGGG
Zscan4 locus-3	TCCCTAGAATACAGTCCTCA	GTAGAATCCTTGATAGTGGG
subtel_chr7	GGGGGTCTTGATACAACTTCAGAA	TGCTGTTTCCCACCTTGACCTGTCC
subtel_chr13	GCACACTTGGTGGGCTAAGAAGATG	TTAAATCCTGACCAAAATGCCTGGC
β-actin	CGTGTGACAAAGCTAATGAGGCTG	CTAAGTTCAGTGTGCTGGGAGTCT

Supplementary Table 5 Primers for bisulfite sequencing

Primer	Forward	Reverse
Ch7 end	GGGGGTTTTGATATAATTTTAGAA	ATAAAAAACAACCCTTAACACATAC
Ch13 end	TTGGTATATTTGGTGGGTTAAGAAG	AATCCACAATTCTCCTTCAAAAATA

Supplementary Table 6 Knockdown sequences

shRNA	Sequence
control shRNA forward	gatccGGCGTTCAATTAGCAGACCATTCAAGAGATGGTCTGCTAATTGAACGCCTTTTTTAAGCTTg
control shRNA reverse	aattcAAGCTTAAAAAAGCGTTCAATTAGCAGACCATCTCTTGAATGGTCTGCTAATTGAACGCCg
Dnmt3b sh-1 forward	gatccGGAGTTGGGTATTAAAGTGTTCAAGAGACACTTTAATACCCAACTCCTTTTTTAAGCTTg
Dnmt3b sh-1 reverse	aattcAAGCTTAAAAAAGGAGTTGGGTATTAAAGTGTCTCTTGAACACTTTAATACCCAACTCCg
Dnmt3b sh-2 forward	gatccGCTTGAAGTAGGTAGTAAGATGTTCAAGAGACATCTTACTACCTACTTCAAGCTTTTTTAAGCTTg
Dnmt3b sh-2 reverse	aattcAAGCTTAAAAAAGCTTGAAGTAGGTAGTAAGATGTCTCTTGAACATCTTACTACCTACTTCAAGCg
Notch4 sh-1 forward	gatccGCCGTTAAGCTCACTTGTCTTTCAAGAGAAGACAAGTGAGCTTAACGGTTTTTTAAGCTTg
Notch4 sh-1 reverse	aattcAAGCTTAAAAAACCGTTAAGCTCACTTGTCTTCTCTTGAAAGACAAGTGAGCTTAACGGCg
Notch4 sh-3 forward	gatccGCTGTGAGATGAACCCAGATTTCAAGAGAATCTGGGTTCATCTCACAGTTTTTTAAGCTTg
Notch4 sh-3 reverse	aattcAAGCTTAAAAAACTGTGAGATGAACCCAGATTCTCTTGAAATCTGGGTTCATCTCACAGCg

Zscan4 sh-1 forward	gatccCAGAAGCCTGGCATTCCCTTTCAAGAGACACTTTAATACCCAACTCCTT TTTTAAGCTTg
Zscan4 sh-1 reverse	aattcAAGCTTAAAAAACAGAAGCCTGGCATTCCCTTCTCTTGAACACTTTAATA CCCAACTCCg
Zscan4 sh-2 forward	gatccGAGTGAATTGCTTTGTGTCTTCAAGAGACACTTTAATACCCAACTCCTT TTTTAAGCTTg
Zscan4 sh-2 reverse	aattcAAGCTTAAAAAAGAGTGAATTGCTTTGTGTCTCTCTTGAACACTTTAATA CCCAACTCCg
